# Heart Failure Readmission Prevention Strategies—A Comparative Review of Medications, Devices, and Other Interventions

**DOI:** 10.3390/jcm14165894

**Published:** 2025-08-21

**Authors:** Remzi Oguz Baris, Corey E. Tabit

**Affiliations:** Section of Cardiology, Department of Medicine, The University of Chicago, Chicago, IL 60637, USA

**Keywords:** heart failure readmissions, heart failure pharmacotherapy, readmission risk scoring

## Abstract

Heart failure readmissions remain a major challenge for healthcare systems, contributing significantly to morbidity, mortality, and increased healthcare costs. Despite advancements in medical and device-based therapies, rehospitalization rates remain high, particularly within the first 30 days of discharge. This review aims to evaluate the primary factors associated with HF readmissions and discuss evidence-based strategies to reduce these rates. The review examines the efficacy of pharmacological therapies and their impact on readmission rates, highlighting key interventions such as diuretics, beta-blockers, ACE inhibitors, ARBs, ARNIs, SGLT2 inhibitors, and intravenous iron supplementation. Additionally, device-based interventions, including CardioMEMS, LVADs, CRT-P/D, ICDs, Furoscix, and the ReDS vest, are critically evaluated for their role in the early detection and management of decompensation. Non-pharmacological strategies are also underscored, such as dietary modifications, exercise, cardiac rehabilitation, and structured follow-up programs. By synthesizing current evidence, this review provides a comprehensive analysis of heart failure readmission factors and proposes multidisciplinary, patient-centered strategies to improve outcomes and reduce hospitalizations.

## 1. Introduction

Heart failure (HF) remains a significant global health challenge affecting approximately 6.7 million Americans over 20 years of age, and the prevalence is expected to rise to 8.5 million Americans by 2030 [1]. Heart failure poses challenges to healthcare systems through hospitalizations and readmissions and is the leading cause of hospitalization in the United States and Europe [2]. Approximately 1 million individuals in the US are admitted with this diagnosis annually [3]. Further, approximately 20–50% of hospitalized heart failure patients are readmitted within 30 days to 1 year of discharge [4]. High expenses for heart failure care lead to financial challenges for patients, which impact adherence to guideline-directed medical therapies. Noncompliance, in turn, leads to poor outcomes, such as worsening symptoms, higher readmission rates, and higher mortality rates [5]. To improve patient wellbeing and decrease the economic burden on the healthcare system, it is important for clinicians to focus on readmission prevention strategies.

In the US, heart failure readmission rates have been key as an indicator for quality assessment. However, this indicator must be carefully interpreted due to various underlying causes of readmissions [6]. These include patient-related noncompliance to treatment, improper follow-up, insufficient prescription of guideline-directed medical therapies, and overreliance on Emergency Departments.

This review will examine readmission-prevention strategies by comparing the efficacy of current medications, the contribution of healthcare system components, and the role of medical devices used for heart failure treatment in the literature.

## 2. Pharmacologics

### 2.1. Diuretics

Diuretics are the main treatment option used to prevent fluid retention. Factors such as drug selection and dosage have a significant impact on readmissions [7]. Among loop diuretics, furosemide, torsemide, and bumetanide are the most commonly used treatment options. These drugs induce diuresis by inhibiting sodium reabsorption in the thicker branch of the loop of Henle [8]. Furosemide, which is widely used in clinical practice, provides an irregular diuretic effect due to its short half-life and bioavailability [9]. In contrast, torsemide has the potential to provide a predictable fluid balance due to its long half-life and more stable bioavailability. In systematic analyses, readmission rates were significantly lower in patients on torsemide [10]. However, in the TRANSFORM-HF randomized trial, torsemide did not result in a mortality benefit vs. furosemide [11].

Thiazide diuretics (e.g., metolazone) are often used as an add-on treatment option to overcome resistance to loop diuretics. Although this combination has a more effective diuresis effect, this more-complicated management strategy may indirectly increase the risk of hospitalization, as it may cause side effects such as hyponatremia, hypokalemia, and acute kidney injury [12] and can lead to challenges with patient compliance.

Aldosterone antagonists (spironolactone, eplerenone) contribute not only to symptom control, but also to the natural history of heart failure. In the RALES and EMPHASIS-HF trials, these drugs were found to be effective in reducing both mortality and hospital readmission rates in patients with NYHA II–IV heart failure. In particular, eplerenone has been found to significantly reduce the risk of hospital readmission even at low symptom severity [13].

Among hospitalized patients, effects between high-dose and low-dose loop diuretic strategies are clinically significant but limited. The use of high-dose intravenous loop diuretics provides faster symptom relief but may lead to temporary deterioration in renal function [9]. Data from observational studies have also shown that high-dose loop diuretic use is associated with worse renal outcomes and higher rehospitalization rates in heart failure patients [14]. On the other hand, although low-dose therapy preserves renal function, it may lead to an increased risk of early rehospitalization if adequate decongestion is not achieved.

Some critical studies have evaluated the effect of diuretic doses on readmission rates. In a multicenter retrospective study, increasing the loop diuretic dose used at discharge in patients with acute heart failure compared to the dose used at home reduced the 30-day all-cause rehospitalization rate to 20%, which was significantly lower than the 37% rate in the group where no dose adjustment was made (adjusted OR = 0.32, *p* = 0.017) [15]. Similarly, in a different study, the 30-day rehospitalization risk was found to be lower in patients whose discharge dose was increased [16]. While important, these retrospective studies should be confirmed with prospective randomized trials.

Diuretics are indispensable agents for the rapid control of heart failure symptoms and reduction of volume overload and are therefore classified as a class I recommendation in both the American (ACC/AHA/HFSA 2022) and European (ESC 2021) heart failure guidelines as one of the cornerstones of symptomatic treatment [17,18]. However, the effect of diuretics on endpoints such as mortality or rehospitalization has not been demonstrated as strongly as that of other heart failure treatments (e.g., SGLT2 inhibitors, ARNIs) in the current literature [18]. In addition, more recent data, such as the DOSE (Diuretic Optimization Strategies Evaluation) study, have shown that the intensity of intravenous diuretic dosage may accelerate symptom relief but does not make a significant difference in long-term clinical outcomes [7].

In summary, individualized diuretic management improves quality of life and decreases rehospitalization rates in heart failure patients by providing optimal decongestion and reducing the risk of complications.

### 2.2. Beta-Blockers

Beta-blockers contribute to the preservation of ventricular function in several different ways, mainly through suppression of sympathetic overactivation, which together lead to reduced heart rate, decreased myocardial oxygen consumption, and attenuation of adverse cardiac remodeling [19,20]. For the treatment of HFrEF, three agents are commonly recommended: carvedilol, bisoprolol, and metoprolol succinate. Carvedilol blocks both β1 and α1 receptors. Through this mechanism of action, it reduces pulse rate and contractility, as well as systemic vascular resistance. Carvedilol treatment has been associated with a significant reduction in readmission rates [21]. Bisoprolol has β1-selective blocking properties and is particularly preferred in patients at low risk of bradycardia and hypotension. It has been shown to significantly reduce both mortality and rehospitalization [22]. Metoprolol succinate is a selective β1 blocker. The use of metoprolol has been found to reduce mortality and hospitalizations, but it has limited effects on vascular resistance, unlike carvedilol [23]. In a comparison of carvedilol with metoprolol tartrate, carvedilol was found to have a significantly greater effect on total mortality and rehospitalization [24]. However, the use of metoprolol tartrate instead of succinate in this study does limit the generalizability of these findings in clinical practice.

With regard to carvedilol, there are limited strong clinical data targeting readmission rates. However, the pre-discharge carvedilol initiation strategy is supported by a series of prospective studies showing a significant reduction in mortality. For example, studies in patients with acute NYHA III–IV heart failure have reported that carvedilol reduces mortality by up to 30% [25,26,27].

Regarding bisoprolol, there are more recent studies based on data regarding 30-day rehospitalization rates, particularly in elderly patient groups. For example, a 2021 prospective cohort study demonstrated a significant reduction in the risk of rehospitalization in patients discharged after oxygen-supported therapy with bisoprolol (HR 0.75, *p* = 0.02), and a 2014 randomized study also found a significant improvement in quality of life (*p* < 0.001) [28].

Dose adjustment of beta-blockers is another important factor. Low doses may not fully achieve protective effects, and optimal titration significantly reduces hospitalization rates [29]. Beta-blocker therapy should generally be started at a low dose and gradually increased. Aggressive titration should be avoided after decompensated heart failure, because it may increase the risk of early readmission [30].

### 2.3. ACE/ARB/ARNI

ACEi inhibits the conversion of angiotensin I to its active form, angiotensin II, thereby reducing systemic vasoconstriction and aldosterone secretion. As a result of these effects, preload and afterload decrease, and cardiac remodeling slows down [31]. Large-scale studies have shown that ACE inhibitors such as enalapril significantly reduce mortality and hospitalizations in HFrEF patients [32,33]. Cough and angioedema are the most important use-limiting side effects.

Angiotensin receptor blockers (ARBs) act by blocking the binding of angiotensin II to AT1 receptors [34]. In HFrEF patients, they are used as an alternative treatment option to ACE inhibitor-intolerant patients. As an ARB, candesartan significantly reduces mortality and hospitalization rates in ACE inhibitor-intolerant patients [35]. However, when compared directly with ACE inhibitors, the effects of ARBs may be less [36].

ARNIs (e.g., sacubitril/valsartan) are a combination of sacubitril, a neprilysin inhibitor, and valsartan. Neprilysin breaks down vasodilator peptides such as natriuretic peptides, bradykinin, and adrenomedullin. Inhibition of this enzyme lowers blood pressure by increasing the levels of these peptides, reducing cardiac stress and fibrosis [37]. A sacubitril/valsartan combination has been shown to significantly reduce HFrEF-related rehospitalization by approximately 21% compared with enalapril due to natriuresis, diuresis, and vasodilation mechanisms of action [38].

In light of the available literature and clinical data, sacubitril/valsartan (ARNI) therapy appears to be superior to other treatment modalities [17,38]. Therefore, current guidelines recommend that ARNI should be initiated as first-line therapy in patients with symptomatic HFrEF [39].

### 2.4. SGLT2 Inhibitors

SGLT2 inhibitors have recently become one of the main therapeutic options in the treatment of heart failure due to their cardiovascular benefits. Drugs such as empagliflozin and dapagliflozin reduce plasma volume by inducing glycosuria, thereby reducing cardiac preload and afterload [40,41]. In large-scale early studies, these agents have been shown to significantly reduce major adverse cardiovascular events and hospitalizations for heart failure [42]. In another randomized controlled trial, SGLT2 inhibitors were also shown to be strongly effective in HFrEF patients without diabetes [43,44].

Studies on dapagliflozin have shown that when added to standard heart failure treatment, it reduces the risk of hospitalization by 30% [43]. The use of empagliflozin was found to significantly reduce the rates of cardiovascular death or heart failure-related hospitalization in HFrEF patients [44]. In both trials, the observed benefit was independent of LVEF. In addition, empagliflozin was also found to significantly reduce hospitalizations in patients with HFpEF [45]. The efficacy of SGLT2 inhibitors is increasingly being emphasized in patients with heart failure with preserved ejection fraction (HFpEF). In the EMPEROR-Preserved study, empagliflozin significantly reduced the risk of the composite primary endpoint of cardiovascular death or hospitalization by 21% compared to placebo in patients with HFpEF (HR 0.79, *p* = 0.001) [45]. Similarly, the DELIVER study also demonstrated that dapagliflozin significantly reduced total heart failure hospitalizations and symptom worsening in the HFpEF population (HR 0.82, *p* < 0.001) [46]. In light of these findings, dapagliflozin is now recognized as an evidence-based treatment option not only for HFrEF but also for HFpEF patients [18].

In addition to their diuretic effects, SGLT2 inhibitors have other effects on patients with heart failure. They directly affect the underlying pathophysiology of the disease by many mechanisms, including lowering intraglomerular pressure, anti-inflammatory effects, improving myocardial metabolism, and increasing insulin sensitivity [41,47].

According to recently published guidelines, SGLT2 inhibitors have been integrated into therapy by adding them to the class I recommendation for prolonging survival and preventing readmissions in symptomatic patients. There is also a growing use for patients with HFpEF [17].

### 2.5. MRA

Mineralocorticoid receptor antagonists (MRAs), especially spironolactone and eplerenone, have an important role in the management of HFrEF patients. By blocking aldosterone receptors, they reduce volume overload and improve cardiac fibrosis and inflammation [13]. Early initiation of MRAs has a positive effect on mortality, quality of life, and survival [48]. Long-term data show that MRAs also prevent repeated readmissions [49].

In the RALES (Randomized Aldactone Evaluation Study) trial, spironolactone reduced all-cause mortality by 30% in patients with NYHA class III–IV symptoms and LVEF below 35% [50]. Other studies have also shown that eplerenone significantly reduced cardiovascular-related mortality and hospitalization rates in patients with left ventricular dysfunction after myocardial infarction [51]. Even in patients with milder symptoms, i.e., NYHA class II patients, eplerenone has been found to have a significant benefit [13]. While foundational trials remain essential, recent evidence adds contemporary relevance and supports their ongoing applicability. An individual patient meta-analysis confirmed that traditional MRAs continue to reduce cardiovascular mortality and heart failure hospitalization in diverse HFrEF populations, including patients with chronic kidney disease, with consistent hazard ratios across multiple subgroups (HR ≈ 0.80) [52].

One of the most important contributions of MRAs is the inhibition of cardiac remodeling by suppressing neurohormonal activity. Thus, it reduces the incidence of ventricular arrhythmias and the risk of sudden cardiac death [53]. However, the risk of treatment-induced hyperkalemia and renal dysfunction should be considered, especially in elderly patients, diabetic individuals, and patients receiving ACE/ARB therapy [54]. The current ACC/AHA/HFSA guideline recommends the use of MRA as a class I indication in patients with HFrEF. Because eplerenone has a lower endocrine side effect profile than spironolactone, it should be used as a first choice in patients with gynecomastia or sexual dysfunction [17]. In addition, new generation non-steroidal MRAs, such as finerenone, are currently under ongoing clinical trials with more selective effects and lower hyperkalemia potential [55].

The FIDELIO-DKD and FIGARO-DKD studies have shown that finerenone improves both cardiovascular and renal outcomes, particularly in patients with diabetes-related chronic kidney disease. In the FIDELIO-DKD study, the rate of hospitalization due to heart failure was significantly lower in patients treated with finerenone compared with placebo (HR 0.78; *p* = 0.0018) [55]. The FIGARO-DKD study similarly confirmed cardiovascular benefit, particularly in patients with heart failure and preserved eGFR levels (HR 0.87; *p* = 0.03) [56]. Based on these results, finerenone is being considered as a new treatment option, particularly in patients with heart failure and concomitant CKD and diabetes [57].

In conclusion, the potential benefits of these agents can be maximized with proper patient selection, regular laboratory monitoring, and careful dose titration. Further studies may refine treatment algorithms by providing clearer long-term results of new-generation MRAs.

### 2.6. Furoscix^®^

Intravenous loop diuretics are often used for symptom and volume load control in heart failure. However, such treatment typically requires hospitalization, which limits its use in the ambulatory setting. Furoscix^®^ is a portable, subcutaneously administered furosemide system. Approved in 2022 by the FDA, this modern treatment method has reduced hospitalization rates while providing diuresis in ambulatory patients [58].

Furoscix is a pump system that infuses 80 mg furosemide subcutaneously over 5 h. It achieves a similar level of efficacy as IV furosemide in a less invasive way [59]. In a prospective study, Furoscix^®^ treatment provided similar diuresis and significantly reduced hospitalization rates in a group of hemodynamically stable patients with an indication for hospitalization [60]. At a 30-day follow-up, hospital readmissions were 30% lower in the Furoscix^®^ group compared with standard IV therapy. In addition, the avoidance of hospitalization has been shown to improve quality of life [61].

While Furoscix^®^ provides a practical treatment option for individuals who have difficulty accessing long-term IV therapy, have chronic volume overload, or have difficult access to hospitals. Its use is not recommended in patients with severe hypoperfusion or shock, and hemodynamic stability of the patient must be ensured.

### 2.7. Intravenous Iron

Iron deficiency is a common but neglected comorbidity in heart failure patients. Approximately 50% of HFrEF patients are iron deficient, and this has a negative impact on quality of life and prognosis [62]. Intravenous iron therapy is becoming more prominent among the treatment options considering the inadequate efficacy of oral iron therapy, such as limited absorption and gastrointestinal intolerance.

In a randomized controlled trial, IV ferric carboxymaltose therapy was shown to improve symptoms and increase functional capacity in iron-deficient HFrEF patients [63]. In a follow-up study, IV iron therapy significantly improved both 6 min walking distance and NYHA functional classification [64]. In the AFFIRM-AHF study, individuals hospitalized with iron deficiency and acute decompensated heart failure were given IV ferric carboxymaltose after stabilization, and after 52 weeks of follow-up, recurrent hospitalizations were reduced by 21% compared with the placebo group [65].

Proactive planning of this treatment in patients at high risk of rehospitalization may not only improve clinical outcomes but also reduce the burden on the healthcare system. IV ferric carboxymaltose therapy in patients with recurrent hospitalizations and reduced functional capacity has been categorized as class IIa in current guidelines [18].

Among the available IV iron preparations, ferric carboxymaltose is the most widely used, although alternatives such as iron sucrose are also available. However, ferric carboxymaltose is mostly preferred in clinical practice, since dose adjustment and frequency of administration vary [66]. Discussions on its effect on long-term mortality are ongoing, and the data to be obtained in ongoing studies will further clarify treatment algorithms.

### 2.8. Inotropes

Inotropic agents are used to maintain perfusion, especially in HFrEF patients. Although these agents provide symptomatic improvement and stabilization, their effect on mortality is still a matter of debate. They are generally indicated in patients with HFrEF who do not respond to conventional therapies and have signs of hypoperfusion [67].

Among the most commonly used intravenous inotropes, dobutamine is a β1 adrenergic receptor agonist, and milrinone works as a phosphodiesterase-3 inhibitor and has both inotropic and vasodilator effects [68]. Although these therapeutic options may improve symptoms in the short term, they require caution in the long term due to the increased risk of arrhythmia and mortality [69]. In terms of hospital readmission, for example, a database analysis showed that patients started on inotropes had higher rates of readmission and death within 30 days [70]. The result of this study suggests that inotropic therapies should be used more as a ‘gateway therapy’. On the other hand, in patients with an INTERMACS profile of 1–3, short-term use of inotropes as a bridge before LVAD or transplantation may be of significant benefit [71]. Although chronic prescription of inotrope therapy in some centers is a major problem, this approach has not been shown to reduce rehospitalization rates. In fact, it is thought that such practices may trigger a “cycle of rehospitalization” [72].

### 2.9. Qishen Yiqi

Qishen Yiqi (QSYQ) is a treatment consisting of four main herbs originating from traditional Chinese medicine. It has long been used as a complementary medicine approach in China. In a randomized clinical trial, a significant increase in left ventricular ejection fraction and a decrease in NT-proBNP levels were observed in the group using QSYQ during a 6-month follow-up. QSYQ has been reported to provide significant clinical benefits in patients with ischemic heart failure, with a 22% reduction in rehospitalization rates [73].

Qishen Yiqi has been found to suppress myocardial remodeling and cardiac fibrosis, especially by regulating PI3K/Akt/mTOR and TGF-β1/Smad signaling pathways. It shows cardioprotective effects with its anti-inflammatory, antioxidant, and antifibrotic properties [74,75]. QSYQ is thought to be more effective when used in addition to standard heart failure treatment modalities. In addition to its positive effect on NYHA functional class, 6 min walking distance, and NT-proBNP levels, studies support that it may also reduce readmissions [76].

The Chinese-centered nature of the patient populations used in the studies requires cautious interpretation regarding universal applicability. Multicenter, international randomized controlled trials are needed to demonstrate the clinical efficacy of QSYQ clearly and to ensure its integration with Western medicine.

### 2.10. Novel Medications

Recently developed pharmacological agents such as vericiguat (sGC stimulator) and omecamtiv mecarbil (cardiac myosin activator) have the potential to reduce rehospitalizations in patients with HFrEF. In the VICTORIA study, vericiguat reduced the risk of cardiovascular death or HF hospitalization by 10% compared with placebo in high-risk symptomatic HFrEF patients (HR 0.90; *p* = 0.02) [77]. In the GALACTIC-HF study, omecamtiv mecarbil reduced the combined risk of HF events or cardiovascular death by 8%; this efficacy was statistically significant in high-risk patients with NYHA class III–IV [78]. However, the current literature emphasizes that additional efficacy data are needed for FDA approval of omecamtiv, while vericiguat is recommended as an adjunct to current standard therapy [79].

## 3. System-Level Interventions

### 3.1. Follow-Up Appointments

Regular follow-up appointments after discharge are a key strategy to reduce the risk of rehospitalization. In particular, hospital appointments within the first 7–14 days following discharge are important for early detection of complications, assessment of treatment compliance, and necessary intervention [80].

In a large retrospective study, cardiology follow-up within the first 7 days was found to significantly reduce 30-day readmissions [81]. Another analysis based on Medicare data reported that patients who followed up within the first week had a 20% lower risk of rehospitalization [82].

Follow-up visits include assessment of parameters such as blood pressure, sudden changes in weight, symptoms, biomarker levels, review of treatment modality, and patient education. This process is a highly effective way of maintaining clinical stability and enabling the patient to better manage their own disease [83]. In addition, a multidisciplinary approach including nurses, dieticians, and pharmacists increases treatment compliance and reduces readmissions [84].

Therefore, guidelines strongly recommend scheduling a follow-up appointment in the first week after discharge. However, a well-coordinated health infrastructure is necessary for follow-up appointments to be effective. Implementation failures have reduced this potential benefit, so hospital follow-up appointment scheduling and patient education should be proactively addressed [85].

### 3.2. Post-Discharge Telephone Calls

Telephone calls stand out as a low-cost and accessible follow-up tool. In the post-discharge period, they contribute to reducing the risk of rehospitalization through patient education, treatment adherence assessment, and early symptom detection [86].

In a randomized controlled trial, patients who received weekly phone calls for 30 days after discharge had a 44% lower readmission rate [87]. These phone calls are mainly conducted by nurses and cardiologists. They focus on topics such as symptomatology, medication adherence, and diet [88].

For telephone interventions to be effective, calls should follow a structured protocol. Random calls or calls focused solely on patient complaints are not an effective intervention tool. Proactive and content-rich conversations increase patient motivation and contribute to clinical stability [89].

In addition, telephone follow-up reduces social isolation and strengthens communication between the patient and the health professional. In some centers, the integration of digital platforms adds visual elements to patient follow-up. It creates a more equitable and effective follow-up mechanism, especially for patients with difficult access to health services [17]. Incorporating telephone calls into routine patient care is increasingly established in heart failure management as an effective, cost-efficient, and sustainable approach to reduce rehospitalization rates.

### 3.3. Heart Failure Disease Management Programs

Heart Failure Disease Management Programs (HF-DMPs) are structured, multi-disciplinary approaches developed to reduce hospitalizations and mortality and improve quality of life. These programs include key components such as patient education, medication adherence monitoring, lifestyle modifications, regular follow-up, telemonitoring, and a multidisciplinary approach. Especially in high-risk individuals, the implementation of these programs is effective in reducing the burden on the healthcare system.

A systematic review demonstrated that heart failure programs with multidisciplinary strategies significantly reduced readmission rates and all-cause mortality [90]. In recent years, it has been shown that rapid optimization of pharmacological therapy in heart failure patients can reduce mortality by more than 60%. This supports the need to extend HF-DMPs to include treatment titration processes [91].

In general, HF-DMPs are now recognized as an extension of evidence-based medicine in the management of HFrEF patients. Guidelines support the feasibility and effectiveness of these programs [17]. However, since success in implementation depends on many factors such as patient motivation, education level of healthcare professionals, and technological infrastructure, HF-DMPs should be structured by taking into account the available resources within each health system.

### 3.4. Community Health Workers

Community health workers (CHWs) are important stakeholders who enhance the effectiveness of community-based health initiatives and act as a link between patients and health services. They increase the access and integration of individuals into the health system in areas such as chronic disease management, health education, and social support. CHWs improve treatment adherence and patient satisfaction, especially in communities with low socioeconomic status [92].

CHWs are also important in the management of chronic diseases such as heart failure through patient follow-up, medication adherence, and early intervention strategies. In one study, a significant reduction in 30-day readmission rates by 89% was found in the group receiving community health worker support in contrast to patients receiving usual care [93].

Community health workers play an active role, not only at the patient level but also at the community level, in implementing public health programs and increasing health literacy. They have also been shown to ease the burden on the health system and increase community trust during the COVID-19 pandemic [94].

Despite the high effectiveness of community health worker programs, their sustainability is at risk due to financial challenges. Health policies need to integrate this component as a permanent rather than temporary solution. It is clear that community health worker programs, strengthened by oversight and sustainable funding mechanisms, are essential for optimizing chronic disease management.

### 3.5. Visiting Nurses

Since heart failure is a chronic and progressive disease, optimal treatment after discharge is important. In this context, the visiting nurses provide close follow-up of patients in the home environment, assess medication compliance, and provide early recognition of symptoms. In a prospective study, it was reported that the readmission rates in the patient group included in the visiting nurse program were less than 10% within 30 days [95]. Visiting nurses’ role in patient education, psychosocial support, and informing family members increases the effectiveness of treatment. Patients receiving home visits were reported to have improved self-care behavior and health literacy, as well as lower 90-day rehospitalization rates [96].

Visiting nurses also have a positive impact on healthcare costs. Heart failure patients with visiting nurses have been found to have 7–10% lower total health expenditures than those who do not receive this service, with a more pronounced difference in the elderly [97]. Incentivizing such supportive methods by CMS and health insurances may be preferable in terms of cost-effectiveness. Studies repeatedly show that nursing services provided at home are effective in reducing rehospitalization rates, especially if implemented in the early post-discharge period.

### 3.6. Cardiologist Consultation in the Emergency Department

Initial interventions in patients presenting to the emergency department with heart failure play a decisive role in the prognosis of the patient. The involvement of a cardiologist at this stage improves both the accuracy of the diagnosis and the outcome of treatment. Especially in patients presenting with atypical symptoms, high comorbidities, and difficulties in making an immediate diagnosis, cardiologist support leads to favorable outcomes [98].

Many studies have shown that early cardiology consultation in the emergency department increases the utilization of appropriate treatment options. In prospective studies, health centers with a cardiologist in the ED had significantly lower readmission rates of 14% for heart failure within 30 days and 57% lower 30-day healthcare costs overall [99]. Importantly, cardiologist consultation in the ED does not increase the length of stay in the ED and does not increase ED congestion [100].

Cardiologists make a significant difference in treatment with appropriate diuretic dose adjustment, advanced imaging decisions, early prognostic assessment, and intensive care referral when necessary. Initiation of the first dose of prognostically favorable agents such as beta-blockers or SGLT2 inhibitors in the emergency department reduces early rehospitalization [101].

According to the guidelines published by the American Heart Association (AHA), the importance of multidisciplinary teams in hospitals is emphasized and cardiology evaluation in the emergency department is recommended as a quality indicator, especially for high-risk patients [17]. Accordingly, in some centers, algorithmic approaches developed by cardiologists are applied in emergency departments. These protocols have been reported to reduce both hospitalization rates and 30-day readmission rates [102].

However, it may not be practical to have a cardiologist always present in every emergency department. Alternative models such as virtual consultation systems and early cardiology outpatient clinic appointments can effectively provide similar support in certain cases. Such approaches can also reduce overall costs to the healthcare system [103].

## 4. Lifestyle

### 4.1. Diet/Nutrition

Appropriate dietary habits in heart failure (HF) patients provide control of congestive symptoms, especially by reducing volume overload. Clinical guidelines emphasize the need to restrict sodium intake, limit fluid intake, and provide balanced energy/micronutrient supplementation in heart failure patients [17].

Sodium restriction is one of the most commonly recommended methods, mainly because excessive sodium intake may increase retention and lead to pulmonary congestion. Although the ideal level of this restriction is controversial, an observational study showed a decrease in rehospitalization rates in patients restricted to less than 2.5 g per day [104]. However, one other study suggested that strict sodium restriction (<2000 mg/day) may have adverse effects on hospital readmission and mortality [17]. For this reason, a target range of 2000–3000 mg/day is considered ideal [105].

Cardiac muscular index is closely related to mortality in heart failure patients. Adequate protein and micronutrient intake are important to maintain cardiac metabolism. Deficiencies of elements such as iron, thiamine, and selenium are also frequently associated with metabolic disturbances [106]. Mediterranean-type diets with low sodium but high nutritional value may be beneficial [107].

Nutrition programs tailored to the individual under the supervision of a dietitian improve patient compliance in the post-discharge period. Since restrictive dietary recommendations may lead to low caloric intake or fluid-electrolyte imbalance, they should be implemented with professional supervision. Nutritional strategies applied in an individualized manner and with a multidisciplinary approach are an effective and sustainable intervention model for the prevention of rehospitalizations.

### 4.2. Exercise

Exercise is one of the evidence-based options to improve the long-term prognosis in the treatment of heart failure. It contributes to increasing functional capacity and improving quality of life, especially in patients with low exertional capacity. The HF-ACTION study evaluated the effect of exercise on morbidity and mortality. In this study, regular aerobic exercise training resulted in a significant reduction in hospitalizations. Over a 12-month follow-up period, a 15% reduction in heart failure-related rehospitalization was observed [108].

Regular exercise has been shown to have many cardiovascular and systemic benefits, including reduced peripheral vascular resistance, improved endothelial function, and lower inflammatory biomarkers [109,110]. There is a growing belief that it provides favorable effects on left ventricular diastolic function and cardiac output in patients in group II–III of the NYHA classification [111].

Patient-based exercise programs are important in clinical practice. In a meta-analysis, cardiac rehabilitation programs were found to significantly reduce hospitalization rates at 6-month follow-up [112]. An individualized approach is very important when recommending exercise in heart failure patients, as caution should be exercised in patients with advanced age, poor diastolic function, or pulmonary hypertension. Current guidelines recommend that exercise programs should be initiated and supervised in patients with hemodynamic stability [17].

### 4.3. Cardiac Rehabilitation

Cardiac rehabilitation is a multidimensional intervention to control symptoms and improve quality of life in individuals with heart failure. Components such as exercise training, patient education, and psychosocial support form a holistic approach to disease management [113]. In a Cochrane review that systematically examined the effect of exercise-based cardiac rehabilitation on heart failure showed that cardiac rehabilitation significantly reduced rehospitalization rates [114]. In patients with low left ventricular ejection fraction, functional capacity has been observed to improve with regular participation in programs.

Considering the psychological effects of heart failure, psychosocial interventions during the rehabilitation process are of great importance. The prevalence of depression and anxiety in heart failure patients has been reported to adversely affect treatment adherence and prognosis [115]. Therefore, components that provide psychological support play an important role in reducing the risk of readmission.

The American Heart Association (AHA) and the American College of Cardiology (ACC) emphasize that cardiac rehabilitation should be a core treatment modality in heart failure disease management. In addition, they suggest that patients referred for cardiac rehabilitation may have better outcomes if they are enrolled in the program within the first 2–4 weeks after discharge [17].

In conclusion, multidisciplinary programs integrating both physiological and psychosocial components of cardiac rehabilitation are considered an effective tool to reduce heart failure rehospitalization rates. The available data strongly supports the potential to alleviate the burden on the healthcare system and improve the patient’s quality of life.

## 5. Devices

### 5.1. ReDS Vest

One of the main reasons for high readmission rates in heart failure is the lack of early detection of congestion. ReDS Vest (Remote Dielectric Sensing) offers a more sensitive and quantitative assessment of volume than traditional methods.

This technology measures the fluid content in the lungs via an electromagnetic wave through the chest wall, with an average range of 20–35% considered normal. It is non-invasive, fast, and portable [116]. It has been reported to objectively detect chest congestion, and correlates well with thoracic CT [117]. With this technology, 90-day readmissions were significantly reduced from 33% to 17% compared with the control group [118].

This device contributes to clinical decision making by objectively demonstrating whether adequate decongestion has been achieved before discharge [119]. However, factors such as the high cost of the device may limit its use in some centers. In addition, pathologies such as interstitial lung diseases affect the measurement results.

In conclusion, the ReDS Vest offers an important innovation in terms of accurate detection of congestion, improving discharge decisions and reducing rehospitalizations. It stands out as a technological solution tool for individualized approaches in heart failure management.

### 5.2. CardioMEMS^®^

The CardioMEMS^®^ HF System is a wireless sensor for pulmonary artery pressure (PAP) monitoring. It is inserted through a catheter into the distal segment of the pulmonary artery and collects PAP data. These data are transferred to the data storage system via an electronic device in the patient’s home and can be monitored by clinicians. Measurements are made non-invasively and can only be measured in a specific position [120].

Pulmonary arterial pressure is a parameter that can indicate volume overload before symptoms develop. In conventional monitoring, late symptoms such as weight gain and dyspnea indicate advanced hemodynamic deterioration. With CardioMEMS^®^, these increases are recognized early, and the physician can intervene to prevent hospitalization [121].

The CHAMPION study evaluated patients with NYHA class III heart failure who had been hospitalized at least once in the last 12 months. In this randomized study, a 37% reduction in readmission rate was found in the group with CardioMEMS^®^ during a follow-up period of 15 months [122]. In the same study, significantly more treatment changes were made based on pulmonary artery pressure compared with the conventional follow-up group. The majority of interventions based on device data (over 70%) were performed in asymptomatic patients, proving their contribution to early intervention and patient stabilization [123].

On the other hand, there are some limitations to the widespread use of the device. The implantation of the device to the patient takes place under catheterization laboratory conditions, and there is a risk of complications such as pulmonary artery perforation, infection, or thrombosis. Furthermore, reimbursement policies of health insurances and cost are important limiting factors [124]. Although CardioMEMS offers promising results in reducing hospital readmission rates, the integration of these technologies into widespread clinical practice faces numerous systemic barriers. Firstly, the high cost of the device and its exclusion from insurance coverage, particularly in countries with limited reimbursement systems, significantly restricts access. Additionally, the implantation of the device requires teams with specialized expertise; the lack of such teams constitutes a significant barrier, especially in smaller hospitals. The follow-up process does not end with device implantation—the data from the device must be continuously monitored, evaluated, and integrated into treatment at centers with the necessary digital infrastructure. This process requires collaboration between trained nurses, heart failure specialists, and clinical staff capable of interpreting technology. However, many healthcare institutions lack the necessary professional workforce or cannot maintain continuity. Additionally, deficiencies in patient education, digital literacy, and care coordination can limit the device’s potential effectiveness. For these reasons, factors such as feasibility, integration into the healthcare system, and professional capacity must be evaluated alongside technological effectiveness [125]. Future studies should be designed to include not only clinical benefits, but also the extent to which the healthcare system can accommodate these technologies, the distribution of trained personnel, continuity of care, and long-term cost analyses.

In conclusion, the CardioMEMS^®^ device is an innovative technology that can detect hemodynamic changes at an early stage, thus enabling individualized treatment. However, its effect on long-term mortality has not been clearly demonstrated; more prospective studies are needed to clarify the long-term outcomes, cost-effectiveness, and effects on mortality.

### 5.3. Pacemakers and ICDs

Implantable devices are widely used in HFrEF patients, both for symptom control and to reduce the risk of sudden cardiac death. Among these devices, pacemakers (especially CRT-P) and implantable cardioverter defibrillators (ICDs) play an important role. Both devices act through different pathophysiological mechanisms, and their impact on readmissions depends on many variables.

Pacemakers with cardiac resynchronization therapy (CRT) are used to correct mechanical failure due to left ventricular dyssynchrony. In patients with QRS duration ≥ 130 ms and LVEF ≤ 35%, CRT devices optimize cardiac output by increasing ventricular synchronization. This contributes to an increase in exercise tolerance, a decline in NYHA class, and a reduction in hospitalization rates due to volume overload [126,127].

On the other hand, ICDs have a proven positive effect in reducing the risk of sudden cardiac death in heart failure patients. ICDs detect ventricular tachycardia/fibrillation and regulate the rhythm by cardiac shock or anti-tachycardic pacing. However, the effect of ICDs on readmissions is more limited, and it has been shown that inappropriate shocks, anxiety, and device-related complications (e.g., lead dislocation, infection) may increase readmissions [128,129].

CRT-P vs. ICD comparisons show mixed results. Some studies have shown that ICD devices reduce hospitalizations more than CRT-P [130]. However, this comparison is meaningful in patients at high risk of ventricular arrhythmias. In older patients with high comorbidity and non-ischemic etiology, CRT-P has been shown to provide a similar benefit with fewer complications [131]. The RAFT trial is important for examining the efficacy of ICDs to reduce hospitalization in NYHA class II patients. In this trial, CRT-D devices were shown to reduce all-cause readmission rates by 20% compared with ICD in patients with reduced ejection fraction and wide QRS complexes [132]. In the RESET-CRT trial, there was no significant difference in mortality and hospitalization between the two treatment groups [133]. Therefore, the treatment modality should be based not only on LVEF or QRS duration but also on patient age, comorbidities, life expectancy, and arrhythmia risk.

As a result, both of these devices have an important role in heart failure management. Pacemakers provide rhythm synchronization and reduce readmissions, whereas ICDs are life-saving and are preferred in patients with high arrhythmia risk. The choice of device should be based on the type of patient; otherwise, devices may lead to readmissions due to complications. A combination strategy of CRT-D is often the preferred strategy for many patients, as it combines the mechanical benefits of CRT with the defibrillation capability of an ICD.

### 5.4. ZOLL Heart Failure Management System

Identifying early decompensation is important for preventing emergency department visits and repeat hospitalizations. The HeartLogic Heart Failure Diagnostic algorithm developed by ZOLL Medical Corporation detects physiologic changes in the preclinical period. This system is integrated into implantable cardioverter defibrillator (ICDs) or cardiac resynchronization therapy defibrillator (CRT-D) devices and continuously monitors parameters such as thoracic impedance, overnight heart rate, and respiratory rate [117].

The main advantage of this innovative system is that it provides a warning before hemodynamics deteriorate. It has been reported that the HeartLogic algorithm can detect heart failure deterioration on average 34 days in advance with a sensitivity of 70%. The low false-positive value is an important factor that increases reliability for clinicians. Clinical interventions based on the alerts provided by this system have been shown to significantly reduce readmission rates compared to traditional methods of patient follow-up [134].

Implementation of such proactive systems, especially in high-risk patient groups requiring close follow-up, positively affects both mortality and the economic burden on the healthcare system. However, effective patient education, telemonitoring support, and multidisciplinary collaboration are necessary for the success of the HeartLogic system. Clinicians’ confidence in the algorithm, the effectiveness of alarm management protocols, and the ability to intervene promptly are crucial to the success of this technology.

### 5.5. LVADs

Left ventricular assist devices (LVADs) are mainly used as a bridge therapy to transplant or recovery, or as a destination treatment in advanced heart failure. Currently, LVADs improve survival, but rehospitalization rates are still high, which directly affects patient quality of life and cost-effectiveness.

Approximately 50% of patients are rehospitalized within the first 6 months following LVAD therapy [135]. The main reasons for these hospitalizations include bleeding, infection, thromboembolism, right ventricular failure, and device-related technical complications. Gastrointestinal (GI) bleeding in particular is a common complication due to the increased risk of angiodysplasia in LVAD devices with nonpulsatile flow mechanisms [136,137]. Elevation of vasoactive biomarkers such as Angiopoietin-2 [138] and tumor necrosis factor [139] may regulate LVAD-related angiodysplasia. Infections are another leading cause of rehospitalization in patients undergoing LVAD implantation and cause serious morbidity in these patients. According to INTERMACS data, the rate of hospitalization secondary to infection within the first year is around 30% [140]. Right ventricular failure following LVAD implantation leads to serious complications in patients and may require intensive care. In addition, anticoagulation management is a key determinant of hospitalization, as fluctuations in INR levels increase the risk of both bleeding and thrombosis [141].

In recent years, magnetically levitated pump systems have been coming to the fore. HeartMate 3 stands out in these systems, reducing both mechanical wear and hemocompatibility problems thanks to its full magnetic levitation technology, resulting in lower rates of thrombosis and gastrointestinal bleeding. In the MOMENTUM 3 study, pump thrombosis rates in patients using the HeartMate 3 fell below 1%, and compared to similar devices, there was a significant reduction in readmission rates and bleeding complications [142,143]. These findings make the HeartMate 3 a preferable option, especially for patients requiring long-term support.

A multidisciplinary approach, remote monitoring, telehealth applications, and anticoagulation protocols have been proposed to reduce rehospitalizations in LVAD patients. However, considering the frequency of complications in this patient group, the success of treatment should be evaluated not only with device implantation but also with perioperative preparation, long-term follow-up, and individualized treatment methods.

## 6. Readmission Risk Scoring

### 6.1. HOSPITAL Risk Score

The HOSPITAL score is a seven-parameter scoring system developed to predict the risk of hospital readmission. This model has the potential to improve outcomes by enabling early intervention and planning discharge goals.

The HOSPITAL score consists of seven factors. These are hemoglobin at discharge (hemoglobin < 12 g/dL), discharge from an oncology service, sodium level at discharge (Na < 135 mmol/L), procedure during the index admission, index type of admission, number of admissions during the last 12 months, and length of stay [144]. These variables determine the individual risk score, and a higher score significantly increases the probability of readmission within 30 days [145].

In heart failure patients, the HOSPITAL risk score has been shown to provide similar accuracy in predicting readmission rates when compared with other scoring models such as the LACE score [146]. This feature provides clinicians with a practical tool for early identification of high-risk patients. Indeed, evidence shows that the HOSPITAL score accurately predicts readmission risk and emphasizes the need for early intervention [145]. Further, the HOSPITAL score retains its predictive value even in patient populations with poor social determinants of health (SDOH)—modification of the score to account for SDOH is not necessary [147].

In conclusion, the use of the HOSPITAL risk score in patient populations at high risk of readmission, such as heart failure, may be an effective way to create individualized discharge plans. This score also allows for measures to be taken and implemented to reduce the economic burden on the healthcare system.

### 6.2. LACE Index

Early identification of high-risk patients, individualized treatment, and follow-up planning is critical to reduce the readmission burden for healthcare systems. The LACE index (Length of stay, Acuity of admission, Comorbidities, Emergency department visits) is a practical and widely used scoring system developed to predict readmissions and early mortality within 30 days. The LACE score is a scoring system with high predictive value, especially in patients with a LACE score ≥ 10, where readmission rates within 30 days exceed 25% [148].

In clinical practice, post-discharge care and treatment plans should be individualized for patients with high scores. For example, measures such as early post-discharge outpatient follow-up (<7 days), home nurse follow-up, phone call symptom control, and telemonitoring have a positive contribution to reducing readmission in high-risk patients [146,149]. However, the LACE score is based only on clinical data and excludes social-behavioral factors such as social support, medication adherence, cognitive status, depression, and nutritional status. Therefore, making decisions based on this score alone, especially in older patients, may lead to underestimation of risk.

### 6.3. RAHF Scale

The RAHF (Readmission After Heart Failure) score is a unique scoring system to determine the risk of early rehospitalization. This score addresses multiple parameters such as demographics (age, gender, race), comorbidities, laboratory values, length of hospital stay, and post-discharge planning.

The RAHF score categorizes patients into risk groups. Those with a score below 12 are classified as low risk, those with a score between 12 and 15 are classified as intermediate risk, and those with a score above 15 are classified as high risk. Rehospitalization rates in these groups were 7.58%, 9.78%, and 12.04%, respectively. Thus, clinicians can decide which patients should be followed more closely at the time of discharge [150]. The RAHF scale has significantly better prediction performance in females compared with males [151].

Integration of the RAHF score can create a powerful decision support system for early post-discharge intervention. However, further large-scale prospective studies in different patient populations are needed.

Although the HOSPITAL, LACE, and RAHF scores are individually validated and widely used to estimate readmission risk in heart failure patients, comparative head-to-head analyses between these tools are notably lacking in the literature. No large-scale, prospective studies to date have evaluated these scoring systems under uniform conditions or across similar patient populations. This presents a major gap in the current evidence base. Future research should aim to directly compare the predictive accuracy, ease of clinical application, and context-specific performance of these tools, particularly in diverse healthcare settings and among different heart failure phenotypes. Such studies would provide critical guidance for clinicians seeking to choose the most appropriate risk stratification strategy.

### 6.4. I NEED HELP

The clinical role of risk stratification tools in heart failure is becoming increasingly important. “I NEED HELP”, developed in this context, recognizes both the severity of the disease and the need for further treatment in advanced heart failure. While not a risk score per se, the “I NEED HELP” criteria [I (need for inotropes), N (NYHA class IV), E (EF < 20%), E (worsening end-organ dysfunction), D (defibrillator shocks for ventricular arrhythmias), H (heart failure hospitalizations), E (escalating diuretic dose), L (low blood-pressure), and P (progressive intolerance of GDMT)] predict readmission and mortality. The presence of these parameters is indicative of advanced heart failure and points to possible hospital readmission [152]. Patients with these criteria should be strongly considered for referral to an advanced heart failure specialist.

The main advantage of this scoring is that it is based on easily accessible and non-invasive laboratory data. In addition, it predicts disease progression, allowing timely and aggressive interventions. This system is an effective tool for identifying patients who are candidates for palliative treatment, in addition to those requiring LVAD or transplantation [153].

It has been observed that mortality and rehospitalization rates increase significantly with increasing number of components in the scoring, and this scoring system has high predictive power, especially in NYHA class III–IV patients and in patients with recurrent hospitalizations [154]. A lower score increases the likelihood of a stable course of the disease, and this group of patients can be followed with medical therapy for a longer period of time.

In conclusion, the I NEED HELP criteria are a powerful clinical tool that can predict the risk of rehospitalization in patients with advanced heart failure by systematic assessment. The importance of treatment planning based on an individual risk profile is further enhanced by a multidisciplinary management approach.

### 6.5. Disparities and Social Determinants of Health

While this review primarily focuses on therapeutic interventions at heart failure readmissions, it is important to mention that these outcomes are influenced by social determinants. Racial and ethnic minorities, individuals with lower socioeconomic status, and patients living in rural or underserved regions face higher rates of readmission. These disparities often arise from limited access to specialized care, medication affordability, and transitional care services.

Addressing these disparities requires systemic efforts to mitigate barriers. Future research should prioritize the development and implementation of modalities designed to enhance equity, ensuring that evidence-based therapies are accessible across diverse populations. Although the detailed exploration of these disparities is beyond the scope of this review, it remains an important focus for future research to achieve reductions in heart failure readmissions on a population-wide scale.

## 7. Pulling It All Together—An Integrated Clinical Workflow

**Figure 1:**
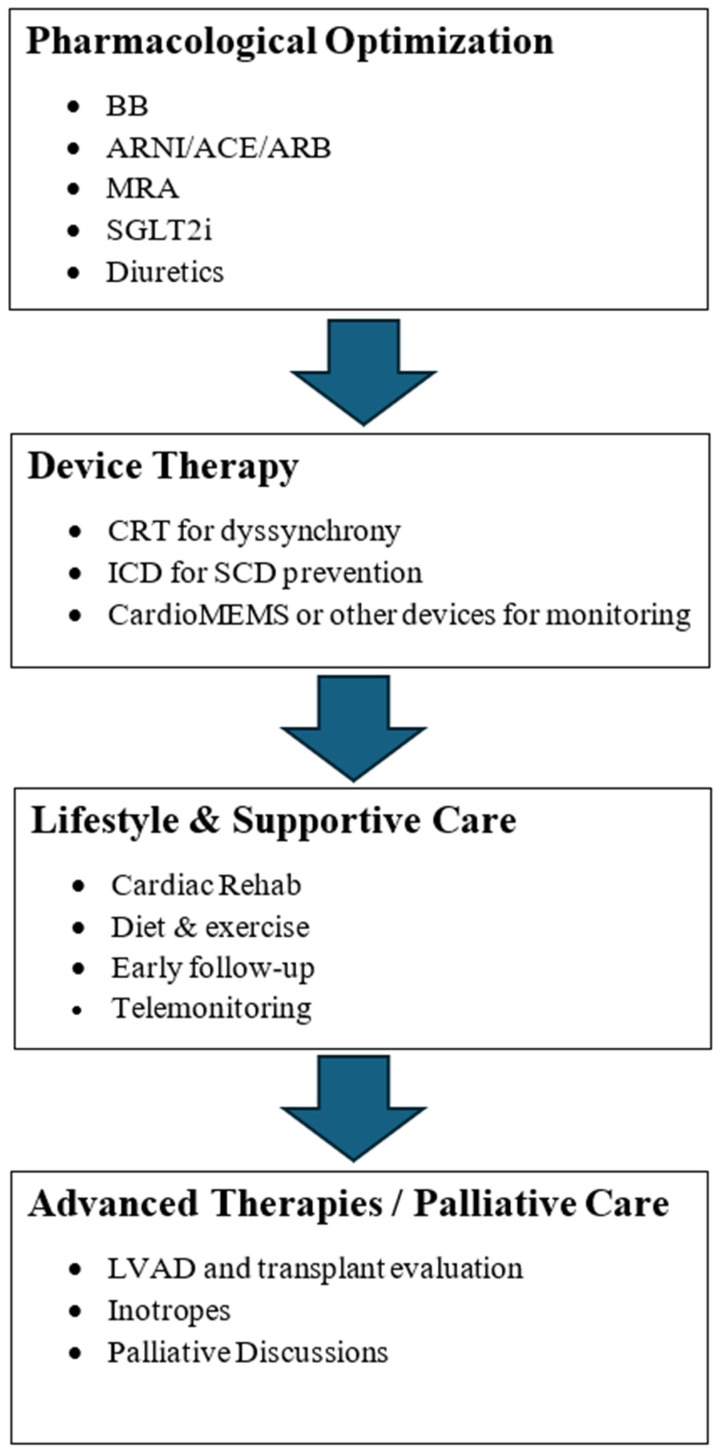
A stepwise approach to heart failure management for readmission reduction.

Effective management to reduce readmission rates necessitates a comprehensive, patient-centered approach that integrates pharmacological therapy, device-based interventions, lifestyle modifications, and system-level strategies. The following workflow (summarized in the Figure 1) reflects real-world practice in a stepwise manner.

### 7.1. Pharmacological Intervention

Initiation of GDMT is the cornerstone of HF management. Beta-blockers, ARNI/ACE/ARBs, MRAs, and SGLT2 inhibitors should be started and titrated to target doses as tolerated. If intolerable side effects occur, alternative GDMT therapies should be considered, and doses should be reevaluated. Diuretics are used for volume management; they should be adjusted to the lowest effective dose to minimize adverse outcomes while maintaining euvolemia. Additional pharmacologic therapies should be considered on an individualized basis.

### 7.2. Device-Based Therapies

Patients with heart failure with reduced ejection fraction (HFrEF) should be systematically evaluated for device therapy. Cardiac resynchronization therapy (CRT) is designed for those with persistent symptoms and electrical dyssynchrony (QRS ≥ 130 ms). Implantable cardioverter defibrillators (ICDs) are considered for prevention of sudden cardiac death. In selected NYHA class III patients with frequent hospitalizations, remote hemodynamic monitoring using devices like CardioMEMS can provide significant improvement in outcomes.

### 7.3. Lifestyle and Supportive Care

Non-pharmacological interventions play a pivotal role in preventing exacerbations. Cardiac rehabilitation programs enhance functional capacity and medication adherence. Dietary sodium and fluid restrictions are reinforced alongside individualized exercise programs. Early post-discharge follow-up visits within 7–14 days are critical for early detection of clinical deteriorations. Telemonitoring interventions, such as phone calls and home visits by specialized nursing teams, ensure ongoing patient engagement and possible early intervention.

### 7.4. Advanced Therapies and Palliative Care Considerations

For patients with advanced HF who remain symptomatic despite optimal therapy, advanced interventions such as left ventricular assist devices (LVADs), inotropes, or heart transplantation should be considered. Simultaneously, timely discussions regarding goals of care and palliative approaches should be initiated to align treatment strategies with patient preferences and quality-of-life priorities.

This integrated workflow emphasizes that reducing HF readmissions is a dynamic and collaborative process, involving strong coordination between interventions tailored to each patient’s clinical trajectory.

## 8. Future Studies

Although the current literature describes numerous interventions aimed at reducing hospital readmissions in heart failure, there are still significant data gaps regarding the comparative efficacy, cost-effectiveness, and feasibility of these approaches. For example, while the PARADIGM-HF study reported a 21% reduction in readmissions with ARNI therapy, and the CHAMPION study reported a 37% reduction with the CardioMEMS device in similar patient populations, these two interventions are associated with different mechanisms, implementation challenges, cost structures, and patient profiles. Therefore, direct comparison of these rates may be misleading. Nevertheless, future randomized controlled trials should enable direct comparison of different interventions to further optimize individualized treatment approaches. Such studies should also include parameters beyond clinical outcomes, such as quality-of-life measures, cost-benefit analyses, and the burden on healthcare systems.

## 9. Summary

Heart failure readmissions remain a multifaceted clinical challenge, deeply related to the pathophysiology of the disease, patient behaviors, and timely medical interventions. This review illustrates that a combination of pharmacologic therapies (Table 1), device-based interventions (Table 2), structured lifestyle modifications (Table 3), and post-discharge planning can significantly reduce hospital readmissions. Key pharmacologic agents such as ARNI, beta blockers, SGLT2 inhibitors, MRAs, and intravenous iron have shown evidence in improving patient stability and reducing the burden of readmissions. Device-based tools like CardioMEMS and ReDS Vest enable earlier detection of deterioration, while Furoscix offers outpatient decongestive therapy, amplifying the importance of proactive and ambulatory care.

Importantly, the success of preventing readmission is linked to the continuum of care. Interventions such as early follow-up appointments, phone calls, and support of visiting nurses or community health workers have demonstrated a beneficial effect in maintaining patient adherence and facilitating early recognition of worsening symptoms. Risk stratification tools like the LACE index, HOSPITAL score, RAHF scale, and I NEED HELP criteria (Table 4) allow detection and prioritization of high-risk patients. Multidisciplinary heart failure disease management programs consolidate these components into guideline-driven care pathways, with proven reductions in readmission rates.

At our center, we have adopted a patient-centered multidisciplinary model of continuous care. Our approach integrates cardiologists, heart failure nurse specialists, clinical pharmacists, dietitians, and social workers into a team that follows each patient from hospitalization through outpatient recovery. Standardized protocols guide the evidence-based therapies, while structured follow-up visits, telemonitoring, and phone calls reinforce early symptom recognition. This coordinated structure also promotes patient engagement, care transitions, and reduction in preventable readmissions. We believe that similar multidisciplinary models can serve as a cornerstone for improving heart failure outcomes.

Practical clinical decisions, such as transitioning from intravenous to subcutaneous diuretics or determining the appropriate timing for palliative care referral, are highly dependent on individual patient factors. These include hemodynamic status, comorbidities, personal goals, and social and logistical circumstances. As such, offering broad clinical recommendations may oversimplify complex care pathways and risk misleading interpretations. Furthermore, the primary objective of this review is to summarize evidence-based strategies for reducing heart failure readmissions, rather than to serve as a clinical guideline. Therefore, detailed recommendations tailored to specific care situations are beyond the scope of this manuscript but are indeed important topics for future focused publications.

In conclusion, the reduction of heart failure readmissions requires a dynamic, patient-centered approach. Institutions that commit to multidisciplinary strategies tailored to patient risk profiles significantly reduce the burden on healthcare systems. Future research must continue to refine these interventions and fill the gaps in implementation across diverse healthcare settings.

## Figures and Tables

**Table 1:** Pharmacological interventions.

Drug Class	Mechanism of Action	Readmission Impact	Level of Evidence (LOE)
**Beta-Blockers**	Suppress sympathetic overactivation, reduce heart rate	Reduces mortality and readmission in HFrEF	A
**ACE Inhibitors**	Inhibit conversion of angiotensin I to II, reduce vasoconstriction	Reduces mortality and hospitalization	A
**ARBs**	Block angiotensin II at AT1 receptor	Alternative to ACEi in intolerant patients	A
**ARNIs**	Neprilysin inhibition increases natriuretic peptides, vasodilation	21% reduction in readmissions	A
**SGLT2 Inhibitors**	Induce glycosuria, reduce preload/afterload	~30% reduction in hospitalization	A
**MRAs**	Aldosterone antagonism, reduce fibrosis and volume overload	~30% reduction in mortality/readmissions	A
**Diuretics**	Inhibits sodium reabsorption in loop of Henle	Symptom relief, limited direct impact on mortality/readmissions	B-R
**Finerenone**	Non-steroidal MRA, selective aldosterone blockade	Reduces HF hospitalization in CKD patients	B-R
**IV Iron**	Corrects iron deficiency, improves functional capacity	21% reduction in rehospitalization	B-R
**Vericiguat**	Soluble guanylate cyclase stimulator	10% reduction in CV death or HF hospitalization	B-R
**Omecamtiv Mecarbil**	Cardiac myosin activator	8% reduction in HF events in high-risk patients	B-R

Footnote: Level of evidence. Level A: High-quality evidence from more than 1 RCT; Meta-analyses of high-quality RCTs; One or more RCTs corroborated by high-quality registry studies. Level B-R: Moderate-quality evidence from 1 or more RCTs; Meta-analyses of moderate-quality RCTs.

**Table 2:** Device-based interventions.

Device	Mechanism	Readmission Impact	Level of Evidence (LOE)
**CRT-P**	Electrical resynchronization for LV dyssynchrony	Reduces HF hospitalization in selected patients	A
**CRT-D**	CRT combined with defibrillation capability	Reduces both sudden cardiac death and readmission	A
**ICD**	Detects VT/VF, provides shock or anti-tachy pacing	Reduces sudden death, limited readmission impact	A
**CardioMEMS**	Pulmonary artery pressure monitoring	37% reduction in HF readmission	B-R
**ReDS Vest**	Non-invasive lung fluid content measurement	90-day readmissions reduced from 33% to 17%	B-R
**LVAD (HeartMate 3)**	Mechanical circulatory support	Reduced pump thrombosis and GI bleeding	B-R
**Furoscix**	Subcutaneous furosemide delivery	30% reduction in rehospitalization	B-R
**ZOLL HeartLogic**	Multi-sensor algorithm integrated into ICD/CRT-D	Alerts 34 days before decompensation	B-NR

Footnote: Level of evidence. Level A: High-quality evidence from more than 1 RCT; Meta-analyses of high-quality RCTs; One or more RCTs corroborated by high-quality registry studies. Level B-R: Moderate-quality evidence from 1 or more RCTs; Meta-analyses of moderate-quality RCTs. Level B-NR: Moderate-quality evidence from 1 or more well-designed, well-executed nonrandomized studies, observational studies, or registry studies; Meta-analyses of such studies.

**Table 3:** System-level and lifestyle interventions.

Intervention	Mechanism/Component	Readmission Impact	Level of Evidence (LOE)
**Cardiac Rehabilitation**	Multidisciplinary exercise and education program	Significant reduction in rehospitalization	A
**Exercise**	Aerobic training and resistance exercises	15% reduction in HF-related hospitalization	A
**Follow-Up Appointments**	Early post-discharge clinic visits within 7–14 days	~20% reduction on 30-day readmissions	B-NR
**Post-Discharge Telephone Calls**	Structured symptom monitoring and adherence checks	44% reduction in readmission rates	B-NR
**Visiting Nurses**	Home visits for assessment, education, and early symptom detection	Rehospitalizations < 10% at 30 days	B-NR
**Community Health Workers**	Patient education and social support	89% reduction in 30-day readmissions	B-NR
**Diet/Nutrition**	Sodium and fluid management, nutritional optimization	Variable, but improved quality of life	B-NR

Footnote: Level of evidence. Level A: High-quality evidence from more than 1 RCT; Meta-analyses of high-quality RCTs; One or more RCTs corroborated by high-quality registry studies. Level B-NR: Moderate-quality evidence from 1 or more well-designed, well-executed nonrandomized studies, observational studies, or registry studies; Meta-analyses of such studies.

**Table 4:** Risk stratification tools.

Tool Name	Parameters Assessed	Purpose & Notes
**HOSPITAL Score**	Hemoglobin, Sodium, Oncology Service, Procedures, Index Admission	Predicts 30-day readmissions, easy to apply
**LACE Index**	Length of Stay, Acuity, Comorbidities, ED Visits	Predicts readmission and mortality risk
**RAHF Scale**	Demographics, Comorbidities, Labs, LOS, Discharge Planning	Tailored to HF readmission prediction
**I NEED HELP**	Inotropes, NYHA IV, EF < 20%, Organ Dysfunction, etc.	Identifies advanced HF, predicts readmission

## Data Availability

No new data were created or analyzed in this study. Data sharing is not applicable to this article.

## References

[1] Bozkurt B., Ahmad T., Alexander K.M., Baker W.L., Bosak K., Breathett K., Fonarow G.C., Heidenreich P., Ho J.E., Hsich E. (2023). Heart Failure Epidemiology and Outcomes Statistics: A Report of the Heart Failure Society of America. J. Card. Fail..

[2] Ambrosy A.P., Fonarow G.C., Butler J., Chioncel O., Greene S.J., Vaduganathan M., Nodari S., Lam C.S.P., Sato N., Shah A.N. (2014). The global health and economic burden of hospitalizations for heart failure: Lessons learned from hospitalized heart failure registries. J. Am. Coll. Cardiol..

[3] Heidenreich P.A., Fonarow G.C., Opsha Y., Sandhu A.T., Sweitzer N.K., Warraich H.J. (2022). Economic Issues in Heart Failure in the United States. J. Card. Fail..

[4] Kimmoun A., Takagi K., Gall E., Ishihara S., Hammoum P., El Beze N., Bourgeois A., Chassard G., Pegorer-Sfes H., Gayat E. (2021). Temporal trends in mortality and readmission after acute heart failure: A systematic review and meta-regression in the past four decades. Eur. J. Heart Fail..

[5] Yu Y., Liu J., Zhang L., Ji R., Su X., Gao Z., Xia S., Li J., Li L. (2024). Perceived Economic Burden, Mortality, and Health Status in Patients with Heart Failure. JAMA Netw. Open.

[6] Fischer C., Lingsma H.F., Marang-van de Mheen P.J., Kringos D.S., Klazinga N.S., Steyerberg E.W. (2014). Is the readmission rate a valid quality indicator? A review of the evidence. PLoS ONE.

[7] Felker G.M., Lee K.L., Bull D.A., Redfield M.M., Stevenson L.W., Goldsmith S.R., LeWinter M.M., Deswal A., Rouleau J.L., Ofili E.O. (2011). Diuretic strategies in patients with acute decompensated heart failure. N. Engl. J. Med..

[8] Ellison D.H. (2001). Diuretic therapy and resistance in congestive heart failure. Cardiology.

[9] Brater D.C. (1998). Diuretic therapy. N. Engl. J. Med..

[10] Teixeira L., Felix N., Navalha D.D.P., Ferreira R., Clemente M.R.C., Madeira T., Nogueira A., Tramujas L. (2024). Torsemide versus Furosemide in the Treatment of Heart Failure: A Systematic Review and Meta-Analysis of Randomized Controlled Trials. Arq. Bras. Cardiol..

[11] Greene S.J., Velazquez E.J., Anstrom K.J., Clare R.M., DeWald T.A., Psotka M.A., Ambrosy A.P., Stevens G.R., Rommel J.J., Alexy T. (2023). Effect of Torsemide Versus Furosemide on Symptoms and Quality of Life Among Patients Hospitalized for Heart Failure: The TRANSFORM-HF Randomized Clinical Trial. Circulation.

[12] Costanzo M.R., Ronco C., Abraham W.T., Agostoni P., Barasch J., Fonarow G.C., Gottlieb S.S., Jaski B.E., Kazory A., Levin A.P. (2017). Extracorporeal Ultrafiltration for Fluid Overload in Heart Failure: Current Status and Prospects for Further Research. J. Am. Coll. Cardiol..

[13] Zannad F., McMurray J.J., Krum H., van Veldhuisen D.J., Swedberg K., Shi H., Vincent J., Pocock S.J., Pitt B., Group E.-H.S. (2011). Eplerenone in patients with systolic heart failure and mild symptoms. N. Engl. J. Med..

[14] Valente M.A., Voors A.A., Damman K., Van Veldhuisen D.J., Massie B.M., O’Connor C.M., Metra M., Ponikowski P., Teerlink J.R., Cotter G. (2014). Diuretic response in acute heart failure: Clinical characteristics and prognostic significance. Eur. Heart J..

[15] Alshibani M., Alshehri S., Alyazidi W., Alnomani A., Almatruk Z., Almeleebia T. (2020). The Impact of Discharged Loop Diuretic Dose to Home Dose on Hospital Readmissions in Patients with Acute Decompensated Heart Failure: A Retrospective Cohort Study. Heart Surg. Forum..

[16] Woodruff A.E., Kelley A.M., Hempel C.A., Loeffler W.J., Echtenkamp C.A., Hassan A.K. (2016). Discharge Diuretic Dose and 30-Day Readmission Rate in Acute Decompensated Heart Failure. Ann. Pharmacother..

[17] Heidenreich P.A., Bozkurt B., Aguilar D., Allen L.A., Byun J.J., Colvin M.M., Deswal A., Drazner M.H., Dunlay S.M., Evers L.R. (2022). 2022 AHA/ACC/HFSA Guideline for the Management of Heart Failure: A Report of the American College of Cardiology/American Heart Association Joint Committee on Clinical Practice Guidelines. Circulation.

[18] McDonagh T.A., Metra M., Adamo M., Gardner R.S., Baumbach A., Bohm M., Burri H., Butler J., Celutkiene J., Chioncel O. (2021). 2021 ESC Guidelines for the diagnosis and treatment of acute and chronic heart failure: Developed by the Task Force for the diagnosis and treatment of acute and chronic heart failure of the European Society of Cardiology (ESC) with the special contribution of the Heart Failure Association (HFA) of the ESC. Eur. Heart J..

[19] McMurray J.J., Pfeffer M.A. (2005). Heart failure. Lancet.

[20] Packer M. (1992). The neurohormonal hypothesis: A theory to explain the mechanism of disease progression in heart failure. J. Am. Coll. Cardiol..

[21] Packer M., Coats A.J., Fowler M.B., Katus H.A., Krum H., Mohacsi P., Rouleau J.L., Tendera M., Castaigne A., Roecker E.B. (2001). Effect of carvedilol on survival in severe chronic heart failure. N. Engl. J. Med..

[22] CIBIS-II Investigators and Committees (1999). The Cardiac Insufficiency Bisoprolol Study II (CIBIS-II): A randomised trial. Lancet.

[23] MERIT-HF Study Group (1999). Effect of metoprolol CR/XL in chronic heart failure: Metoprolol CR/XL Randomised Intervention Trial in Congestive Heart Failure (MERIT-HF). Lancet.

[24] Poole-Wilson P.A., Swedberg K., Cleland J.G., Di Lenarda A., Hanrath P., Komajda M., Lubsen J., Lutiger B., Metra M., Remme W.J. (2003). Comparison of carvedilol and metoprolol on clinical outcomes in patients with chronic heart failure in the Carvedilol or Metoprolol European Trial (COMET): Randomised controlled trial. Lancet.

[25] Fonarow G.C., Abraham W.T., Albert N.M., Stough W.G., Gheorghiade M., Greenberg B.H., O’Connor C.M., Sun J.L., Yancy C., Young J.B. (2007). Carvedilol use at discharge in patients hospitalized for heart failure is associated with improved survival: An analysis from Organized Program to Initiate Lifesaving Treatment in Hospitalized Patients with Heart Failure (OPTIMIZE-HF). Am. Heart J..

[26] O’Connor C.M., Abraham W.T., Albert N.M., Clare R., Gattis Stough W., Gheorghiade M., Greenberg B.H., Yancy C.W., Young J.B., Fonarow G.C. (2008). Predictors of mortality after discharge in patients hospitalized with heart failure: An analysis from the Organized Program to Initiate Lifesaving Treatment in Hospitalized Patients with Heart Failure (OPTIMIZE-HF). Am. Heart J..

[27] Gattis W.A., O’Connor C.M., Gallup D.S., Hasselblad V., Gheorghiade M. (2004). Predischarge initiation of carvedilol in patients hospitalized for decompensated heart failure: Results of the Initiation Management Predischarge: Process for Assessment of Carvedilol Therapy in Heart Failure (IMPACT-HF) trial. J. Am. Coll. Cardiol..

[28] Bhatia V., Bajaj N.S., Sanam K., Hashim T., Morgan C.J., Prabhu S.D., Fonarow G.C., Deedwania P., Butler J., Carson P. (2015). Beta-blocker Use and 30-day All-cause Readmission in Medicare Beneficiaries with Systolic Heart Failure. Am. J. Med..

[29] Fiuzat M., Wojdyla D., Kitzman D., Fleg J., Keteyian S.J., Kraus W.E., Pina I.L., Whellan D., O’Connor C.M. (2012). Relationship of beta-blocker dose with outcomes in ambulatory heart failure patients with systolic dysfunction: Results from the HF-ACTION (Heart Failure: A Controlled Trial Investigating Outcomes of Exercise Training) trial. J. Am. Coll. Cardiol..

[30] Harrington J., Rao V.N., Leyva M., Oakes M., Mentz R.J., Bosworth H.B., Pagidipati N.J. (2024). Improving Guideline-Directed Medical Therapy for Patients with Heart Failure with Reduced Ejection Fraction: A Review of Implementation Strategies. J. Card. Fail..

[31] Francis G.S. (2001). Pathophysiology of chronic heart failure. Am. J. Med..

[32] Investigators S., Yusuf S., Pitt B., Davis C.E., Hood W.B., Cohn J.N. (1992). Effect of enalapril on mortality and the development of heart failure in asymptomatic patients with reduced left ventricular ejection fractions. N. Engl. J. Med..

[33] Group C.T.S. (1987). Effects of enalapril on mortality in severe congestive heart failure. Results of the Cooperative North Scandinavian Enalapril Survival Study (CONSENSUS). N. Engl. J. Med..

[34] Yusuf S., Pfeffer M.A., Swedberg K., Granger C.B., Held P., McMurray J.J., Michelson E.L., Olofsson B., Ostergren J., Investigators C. (2003). Effects of candesartan in patients with chronic heart failure and preserved left-ventricular ejection fraction: The CHARM-Preserved Trial. Lancet.

[35] Granger C.B., McMurray J.J., Yusuf S., Held P., Michelson E.L., Olofsson B., Ostergren J., Pfeffer M.A., Swedberg K., Investigators C. (2003). Effects of candesartan in patients with chronic heart failure and reduced left-ventricular systolic function intolerant to angiotensin-converting-enzyme inhibitors: The CHARM-Alternative trial. Lancet.

[36] Tai C., Gan T., Zou L., Sun Y., Zhang Y., Chen W., Li J., Zhang J., Xu Y., Lu H. (2017). Effect of angiotensin-converting enzyme inhibitors and angiotensin II receptor blockers on cardiovascular events in patients with heart failure: A meta-analysis of randomized controlled trials. BMC Cardiovasc. Disord..

[37] McMurray J.J., Packer M., Desai A.S., Gong J., Lefkowitz M.P., Rizkala A.R., Rouleau J.L., Shi V.C., Solomon S.D., Swedberg K. (2014). Angiotensin-neprilysin inhibition versus enalapril in heart failure. N. Engl. J. Med..

[38] Solomon S.D., McMurray J.J.V., Anand I.S., Ge J., Lam C.S.P., Maggioni A.P., Martinez F., Packer M., Pfeffer M.A., Pieske B. (2019). Angiotensin-Neprilysin Inhibition in Heart Failure with Preserved Ejection Fraction. N. Engl. J. Med..

[39] Yancy C.W., Jessup M., Bozkurt B., Butler J., Casey D.E., Jr Colvin M.M., Drazner M.H., Filippatos G.S., Fonarow G.C., Givertz M.M. (2017). 2017 ACC/AHA/HFSA Focused Update of the 2013 ACCF/AHA Guideline for the Management of Heart Failure: A Report of the American College of Cardiology/American Heart Association Task Force on Clinical Practice Guidelines and the Heart Failure Society of America. Circulation.

[40] Zinman B., Wanner C., Lachin J.M., Fitchett D., Bluhmki E., Hantel S., Mattheus M., Devins T., Johansen O.E., Woerle H.J. (2015). Empagliflozin, Cardiovascular Outcomes, and Mortality in Type 2 Diabetes. N. Engl. J. Med..

[41] Verma S., McMurray J.J.V. (2018). SGLT2 inhibitors and mechanisms of cardiovascular benefit: A state-of-the-art review. Diabetologia.

[42] Wiviott S.D., Raz I., Bonaca M.P., Mosenzon O., Kato E.T., Cahn A., Silverman M.G., Zelniker T.A., Kuder J.F., Murphy S.A. (2019). Dapagliflozin and Cardiovascular Outcomes in Type 2 Diabetes. N. Engl. J. Med..

[43] McMurray J.J.V., Solomon S.D., Inzucchi S.E., Kober L., Kosiborod M.N., Martinez F.A., Ponikowski P., Sabatine M.S., Anand I.S., Belohlavek J. (2019). Dapagliflozin in Patients with Heart Failure and Reduced Ejection Fraction. N. Engl. J. Med..

[44] Packer M., Anker S.D., Butler J., Filippatos G., Pocock S.J., Carson P., Januzzi J., Verma S., Tsutsui H., Brueckmann M. (2020). Cardiovascular and Renal Outcomes with Empagliflozin in Heart Failure. N. Engl. J. Med..

[45] Anker S.D., Butler J., Filippatos G., Ferreira J.P., Bocchi E., Bohm M., Brunner-La Rocca H.P., Choi D.J., Chopra V., Chuquiure-Valenzuela E. (2021). Empagliflozin in Heart Failure with a Preserved Ejection Fraction. N. Engl. J. Med..

[46] Solomon S.D., McMurray J.J.V., Claggett B., de Boer R.A., DeMets D., Hernandez A.F., Inzucchi S.E., Kosiborod M.N., Lam C.S.P., Martinez F. (2022). Dapagliflozin in Heart Failure with Mildly Reduced or Preserved Ejection Fraction. N. Engl. J. Med..

[47] Lopaschuk G.D., Verma S. (2020). Mechanisms of Cardiovascular Benefits of Sodium Glucose Co-Transporter 2 (SGLT2) Inhibitors: A State-of-the-Art Review. JACC Basic Transl. Sci..

[48] Savarese G., Lund L.H. (2017). Global Public Health Burden of Heart Failure. Card. Fail. Rev..

[49] Stienen S., Ferreira J.P., Vincent J., Busselen M., Li B., McMurray J.J.V., Pitt B., Girerd N., Rossignol P., Zannad F. (2019). Estimated Long-Term Survival with Eplerenone. J. Am. Coll. Cardiol..

[50] Pitt B., Zannad F., Remme W.J., Cody R., Castaigne A., Perez A., Palensky J., Wittes J. (1999). The effect of spironolactone on morbidity and mortality in patients with severe heart failure. Randomized Aldactone Evaluation Study Investigators. N. Engl. J. Med..

[51] Pitt B., Remme W., Zannad F., Neaton J., Martinez F., Roniker B., Bittman R., Hurley S., Kleiman J., Gatlin M. (2003). Eplerenone, a selective aldosterone blocker, in patients with left ventricular dysfunction after myocardial infarction. N. Engl. J. Med..

[52] Jhund P.S., Talebi A., Henderson A.D., Claggett B.L., Vaduganathan M., Desai A.S., Lam C.S.P., Pitt B., Senni M., Shah S.J. (2024). Mineralocorticoid receptor antagonists in heart failure: An individual patient level meta-analysis. Lancet.

[53] Barrera-Chimal J., Bonnard B., Jaisser F. (2022). Roles of Mineralocorticoid Receptors in Cardiovascular and Cardiorenal Diseases. Annu. Rev. Physiol..

[54] Juurlink D.N., Mamdani M.M., Lee D.S., Kopp A., Austin P.C., Laupacis A., Redelmeier D.A. (2004). Rates of hyperkalemia after publication of the Randomized Aldactone Evaluation Study. N. Engl. J. Med..

[55] Bakris G.L., Agarwal R., Anker S.D., Pitt B., Ruilope L.M., Rossing P., Kolkhof P., Nowack C., Schloemer P., Joseph A. (2020). Effect of Finerenone on Chronic Kidney Disease Outcomes in Type 2 Diabetes. N. Engl. J. Med..

[56] Pitt B., Filippatos G., Agarwal R., Anker S.D., Bakris G.L., Rossing P., Joseph A., Kolkhof P., Nowack C., Schloemer P. (2021). Cardiovascular Events with Finerenone in Kidney Disease and Type 2 Diabetes. N. Engl. J. Med..

[57] Nugroho P. (2025). Improving Renal Protection in Chronic Kidney Disease Associated with Type 2 Diabetes: The Role of Finerenone. Endocr. Metab. Immune Disord. Drug Targets.

[58] Dahiya G., Bensimhon D., Goodwin M.M., Mohr J.F., Alexy T. (2022). From Oral to Subcutaneous Furosemide: The Road to Novel Opportunities to Manage Congestion. Struct. Heart.

[59] Osmanska J., Petrie M.C., Docherty K.F., Lee M.M.Y., McMurray J.J.V., Campbell R.T. (2025). Subcutaneous furosemide in heart failure: A systematic review. Eur. Heart J. Cardiovasc. Pharmacother..

[60] Konstam M.A., Massaro J., Dhingra R., Walsh M., Ordway L., Pursley M.S., McLean D.S., Saha S., Close N., Konstam J.M. (2024). Avoiding Treatment in Hospital with Subcutaneous Furosemide for Worsening Heart Failure: A Pilot Study (AT HOME-HF). JACC Heart Fail..

[61] Ellison D.H., Felker G.M. (2017). Diuretic Treatment in Heart Failure. N. Engl. J. Med..

[62] Cohen-Solal A., Leclercq C., Deray G., Lasocki S., Zambrowski J.J., Mebazaa A., de Groote P., Damy T., Galinier M. (2014). Iron deficiency: An emerging therapeutic target in heart failure. Heart.

[63] Anker S.D., Comin Colet J., Filippatos G., Willenheimer R., Dickstein K., Drexler H., Luscher T.F., Bart B., Banasiak W., Niegowska J. (2009). Ferric carboxymaltose in patients with heart failure and iron deficiency. N. Engl. J. Med..

[64] Ponikowski P., van Veldhuisen D.J., Comin-Colet J., Ertl G., Komajda M., Mareev V., McDonagh T., Parkhomenko A., Tavazzi L., Levesque V. (2015). Beneficial effects of long-term intravenous iron therapy with ferric carboxymaltose in patients with symptomatic heart failure and iron deficiencydagger. Eur. Heart J..

[65] Ponikowski P., Kirwan B.A., Anker S.D., McDonagh T., Dorobantu M., Drozdz J., Fabien V., Filippatos G., Gohring U.M., Keren A. (2020). Ferric carboxymaltose for iron deficiency at discharge after acute heart failure: A multicentre, double-blind, randomised, controlled trial. Lancet.

[66] Ambrosy A.P., von Haehling S., Kalra P.R., Court E., Bhandari S., McDonagh T., Cleland J.G.F. (2021). Safety and Efficacy of Intravenous Ferric Derisomaltose Compared to Iron Sucrose for Iron Deficiency Anemia in Patients with Chronic Kidney Disease with and without Heart Failure. Am. J. Cardiol..

[67] Guarracino F., Zima E., Pollesello P., Masip J. (2020). Short-term treatments for acute cardiac care: Inotropes and inodilators. Eur. Heart J. Suppl..

[68] Biswas S., Malik A.H., Bandyopadhyay D., Gupta R., Goel A., Briasoulis A., Fonarow G.C., Lanier G.M., Naidu S.S. (2023). Meta-analysis Comparing the Efficacy of Dobutamine Versus Milrinone in Acute Decompensated Heart Failure and Cardiogenic Shock. Curr. Probl. Cardiol..

[69] Felker G.M., Mentz R.J., Adams K.F., Cole R.T., Egnaczyk G.F., Patel C.B., Fiuzat M., Gregory D., Wedge P., O’Connor C.M. (2015). Tolvaptan in Patients Hospitalized with Acute Heart Failure: Rationale and Design of the TACTICS and the SECRET of CHF Trials. Circ. Heart Fail..

[70] Adams K.F., Jr Fonarow G.C., Emerman C.L., LeJemtel T.H., Costanzo M.R., Abraham W.T., Berkowitz R.L., Galvao M., Horton D.P., Committee A.S.A. (2005). Characteristics and outcomes of patients hospitalized for heart failure in the United States: Rationale, design, and preliminary observations from the first 100,000 cases in the Acute Decompensated Heart Failure National Registry (ADHERE). Am. Heart J..

[71] Slaughter M.S., Rogers J.G., Milano C.A., Russell S.D., Conte J.V., Feldman D., Sun B., Tatooles A.J., Delgado R.M., Long J.W. (2009). Advanced heart failure treated with continuous-flow left ventricular assist device. N. Engl. J. Med..

[72] Hauptman P.J., Mikolajczak P., George A., Mohr C.J., Hoover R., Swindle J., Schnitzler M.A. (2006). Chronic inotropic therapy in end-stage heart failure. Am. Heart J..

[73] He X., Jiang Y., Li S., Liu D., Li Z., Han X., Zhang X., Dong X., Liu H., Huang J. (2023). Efficacy and Safety of QiShen YiQi Dripping Pills in the Treatment of Coronary Heart Disease Complicating Chronic Heart Failure (Syndrome of Qi Deficiency with Blood Stasis): Study Protocol for a Randomized, Placebo-Controlled, Double-Blind and Multi-Centre Phase II Clinical Trial. Int. J. Gen. Med..

[74] Li M., Wang Y., Qi Z., Yuan Z., Lv S., Zheng Y., Yan Z., Wang M., Fu H., Fan X. (2022). QishenYiqi dripping pill protects against myocardial ischemia/reperfusion injury via suppressing excessive autophagy and NLRP3 inflammasome based on network pharmacology and experimental pharmacology. Front. Pharmacol..

[75] Anwaier G., Xie T.T., Pan C.S., Li A.Q., Yan L., Wang D., Chen F.K., Weng D.Z., Sun K., Chang X. (2022). QiShenYiQi Pill Ameliorates Cardiac Fibrosis After Pressure Overload-Induced Cardiac Hypertrophy by Regulating FHL2 and the Macrophage RP S19/TGF-beta1 Signaling Pathway. Front. Pharmacol..

[76] Chen W., Chen J., Wang Y., Yan J., Yan X., Wang D., Liu Y. (2022). The role of Qishen Yiqi dripping pills in treating chronic heart failure: An overview of systematic reviews and meta-analyses. Front. Cardiovasc. Med..

[77] Armstrong P.W., Pieske B., Anstrom K.J., Ezekowitz J., Hernandez A.F., Butler J., Lam C.S.P., Ponikowski P., Voors A.A., Jia G. (2020). Vericiguat in Patients with Heart Failure and Reduced Ejection Fraction. N. Engl. J. Med..

[78] Teerlink J.R., Diaz R., Felker G.M., McMurray J.J.V., Metra M., Solomon S.D., Adams K.F., Anand I., Arias-Mendoza A., Biering-Sorensen T. (2020). Omecamtiv mecarbil in chronic heart failure with reduced ejection fraction: GALACTIC-HF baseline characteristics and comparison with contemporary clinical trials. Eur. J. Heart Fail..

[79] Lavalle C., Mariani M.V., Severino P., Palombi M., Trivigno S., D’Amato A., Silvetti G., Pierucci N., Di Lullo L., Chimenti C. (2024). Efficacy of Modern Therapies for Heart Failure with Reduced Ejection Fraction in Specific Population Subgroups: A Systematic Review and Network Meta-Analysis. Cardiorenal Med..

[80] Albert N.M. (2016). A systematic review of transitional-care strategies to reduce rehospitalization in patients with heart failure. Heart Lung..

[81] Hernandez A.F., Greiner M.A., Fonarow G.C., Hammill B.G., Heidenreich P.A., Yancy C.W., Peterson E.D., Curtis L.H. (2010). Relationship between early physician follow-up and 30-day readmission among Medicare beneficiaries hospitalized for heart failure. JAMA.

[82] Dharmarajan K., Hsieh A.F., Lin Z., Bueno H., Ross J.S., Horwitz L.I., Barreto-Filho J.A., Kim N., Bernheim S.M., Suter L.G. (2013). Diagnoses and timing of 30-day readmissions after hospitalization for heart failure, acute myocardial infarction, or pneumonia. JAMA.

[83] John M., Eisenberg Center for Clinical Decisions and Communications Science (2007). Transitional Care Interventions to Prevent Read-missions for People with Heart Failure. Comparative Effectiveness Review Summary Guides for Clinicians.

[84] Wadhera R.K., Yeh R.W., Maddox K.E.J. (2019). The Hospital Readmissions Reduction Program—Time for a Reboot. N. Engl. J. Med..

[85] Bradley E.H., Curry L., Horwitz L.I., Sipsma H., Wang Y., Walsh M.N., Goldmann D., White N., Pina I.L., Krumholz H.M. (2013). Hospital strategies associated with 30-day readmission rates for patients with heart failure. Circ. Cardiovasc. Qual. Outcomes.

[86] Feltner C., Jones C.D., Cene C.W., Zheng Z.J., Sueta C.A., Coker-Schwimmer E.J., Arvanitis M., Lohr K.N., Middleton J.C., Jonas D.E. (2014). Transitional care interventions to prevent readmissions for persons with heart failure: A systematic review and meta-analysis. Ann. Intern. Med..

[87] Koelling T.M., Johnson M.L., Cody R.J., Aaronson K.D. (2005). Discharge education improves clinical outcomes in patients with chronic heart failure. Circulation.

[88] Clark R.A., Inglis S.C., McAlister F.A., Cleland J.G., Stewart S. (2007). Telemonitoring or structured telephone support programmes for patients with chronic heart failure: Systematic review and meta-analysis. BMJ.

[89] Inglis S.C., Clark R.A., McAlister F.A., Ball J., Lewinter C., Cullington D., Stewart S., Cleland J.G. (2010). Structured telephone support or telemonitoring programmes for patients with chronic heart failure. Cochrane Database Syst. Rev..

[90] McAlister F.A., Stewart S., Ferrua S., McMurray J.J. (2004). Multidisciplinary strategies for the management of heart failure patients at high risk for admission: A systematic review of randomized trials. J. Am. Coll. Cardiol..

[91] Mebazaa A., Davison B., Chioncel O., Cohen-Solal A., Diaz R., Filippatos G., Metra M., Ponikowski P., Sliwa K., Voors A.A. (2022). Safety, tolerability and efficacy of up-titration of guideline-directed medical therapies for acute heart failure (STRONG-HF): A multinational, open-label, randomised, trial. Lancet.

[92] Kim K., Choi J.S., Choi E., Nieman C.L., Joo J.H., Lin F.R., Gitlin L.N., Han H.R. (2016). Effects of Community-Based Health Worker Interventions to Improve Chronic Disease Management and Care Among Vulnerable Populations: A Systematic Review. Am. J. Public Health.

[93] Vohra A.S., Chua R.F.M., Besser S.A., Alcain C.F., Basnet S., Battle B., Coplan M.J., Liao J.K., Tabit C.E. (2020). Community Health Workers Reduce Rehospitalizations and Emergency Department Visits for Low-Socioeconomic Urban Patients with Heart Failure. Crit. Pathw. Cardiol..

[94] Ballard M., Bancroft E., Nesbit J., Johnson A., Holeman I., Foth J., Rogers D., Yang J., Nardella J., Olsen H. (2020). Prioritising the role of community health workers in the COVID-19 response. BMJ Glob. Health.

[95] Shabani F., Maleki M., Noohi F., Taghavi S., Khalili Y., Shahboulaghi F.M., Nayeri N.D., Amin A., Nakhaei Z., Naderi N. (2022). Effect of Home Care Program on Re-hospitalization in Advanced Heart Failure: A Clinical Trial. Iran. J. Nurs. Midwifery Res..

[96] Sterling M.R., Kern L.M., Safford M.M., Jones C.D., Feldman P.H., Fonarow G.C., Sheng S., Matsouaka R.A., DeVore A.D., Lytle B. (2020). Home Health Care Use and Post-Discharge Outcomes After Heart Failure Hospitalizations. JACC Heart Fail..

[97] National Bureau Economic Research (2015). Medical Spending of the Elderly. The Bulletin on Retirement and Disability (BRD) and the Bulletin on Health.

[98] Saxena F.E., Bierman A.S., Glazier R.H., Wang X., Guan J., Lee D.S., Stukel T.A. (2022). Association of Early Physician Follow-up with Readmission Among Patients Hospitalized for Acute Myocardial Infarction, Congestive Heart Failure, or Chronic Obstructive Pulmonary Disease. JAMA Netw. Open.

[99] Tabit C.E., Coplan M.J., Spencer K.T., Alcain C.F., Spiegel T., Vohra A.S., Adelman D., Liao J.K., Sanghani R.M. (2017). Cardiology Consultation in the Emergency Department Reduces Re-hospitalizations for Low-Socioeconomic Patients with Acute Decompensated Heart Failure. Am. J. Med..

[100] Spiegel T.F., Wassermann T.B., Neumann N., Coplan M.J., Spencer K.T., Adelman D., Sanghani R.M., Tabit C.E. (2018). A clinical pathway for heart failure reduces admissions from the ED without increasing congestion in the ED. Am. J. Emerg. Med..

[101] Swat S.A., Xu H., Allen L.A., Greene S.J., DeVore A.D., Matsouaka R.A., Goyal P., Peterson P.N., Hernandez A.F., Krumholz H.M. (2023). Opportunities and Achievement of Medication Initiation Among Inpatients with Heart Failure with Reduced Ejection Fraction. JACC Heart Fail..

[102] Hejjaji V., Scholes A., Kennedy K., Sperry B., Khariton Y., Dean E., Lee D.S., Spertus J.A. (2020). Systemizing the Evaluation of Acute Heart Failure in the Emergency Department: A Quality Improvement Initiative. Circ. Cardiovasc. Qual. Outcomes.

[103] Metra M., Adamo M., Tomasoni D., Mebazaa A., Bayes-Genis A., Abdelhamid M., Adamopoulos S., Anker S.D., Bauersachs J., Belenkov Y. (2023). Pre-discharge and early post-discharge management of patients hospitalized for acute heart failure: A scientific statement by the Heart Failure Association of the ESC. Eur. J. Heart Fail..

[104] Zhou K., Alemayehu W., Rathwell S., McAlister F.A., Ross H., Escobedo J., Saldarriaga C., Colin-Ramirez E., Macdonald P., Arcand J. (2025). The relationship of diuretics and dietary sodium in patients with heart failure: An analysis of the SODIUM-HF trial. Am. Heart J..

[105] Colin-Ramirez E., Sepehrvand N., Rathwell S., Ross H., Escobedo J., Macdonald P., Troughton R., Saldarriaga C., Lanas F., Doughty R. (2023). Sodium Restriction in Patients with Heart Failure: A Systematic Review and Meta-Analysis of Randomized Clinical Trials. Circ. Heart Fail..

[106] Soukoulis V., Dihu J.B., Sole M., Anker S.D., Cleland J., Fonarow G.C., Metra M., Pasini E., Strzelczyk T., Taegtmeyer H. (2009). Micronutrient deficiencies an unmet need in heart failure. J. Am. Coll. Cardiol..

[107] Miro O., Estruch R., Martin-Sanchez F.J., Gil V., Jacob J., Herrero-Puente P., Herrera Mateo S., Aguirre A., Andueza J.A., Llorens P. (2018). Adherence to Mediterranean Diet and All-Cause Mortality After an Episode of Acute Heart Failure: Results of the MEDIT-AHF Study. JACC Heart Fail..

[108] O’Connor C.M., Whellan D.J., Lee K.L., Keteyian S.J., Cooper L.S., Ellis S.J., Leifer E.S., Kraus W.E., Kitzman D.W., Blumenthal J.A. (2009). Efficacy and safety of exercise training in patients with chronic heart failure: HF-ACTION randomized controlled trial. JAMA.

[109] Hambrecht R., Fiehn E., Weigl C., Gielen S., Hamann C., Kaiser R., Yu J., Adams V., Niebauer J., Schuler G. (1998). Regular physical exercise corrects endothelial dysfunction and improves exercise capacity in patients with chronic heart failure. Circulation.

[110] Smart N., Marwick T.H. (2004). Exercise training for patients with heart failure: A systematic review of factors that improve mortality and morbidity. Am. J. Med..

[111] Taylor R.S., Sagar V.A., Davies E.J., Briscoe S., Coats A.J., Dalal H., Lough F., Rees K., Singh S. (2014). Exercise-based rehabilitation for heart failure. Cochrane Database Syst. Rev..

[112] Bozkurt B., Fonarow G.C., Goldberg L.R., Guglin M., Josephson R.A., Forman D.E., Lin G., Lindenfeld J., O’Connor C., Panjrath G. (2021). Cardiac Rehabilitation for Patients with Heart Failure: JACC Expert Panel. J. Am. Coll. Cardiol..

[113] Thomas R.J., Beatty A.L., Beckie T.M., Brewer L.C., Brown T.M., Forman D.E., Franklin B.A., Keteyian S.J., Kitzman D.W., Regensteiner J.G. (2019). Home-Based Cardiac Rehabilitation: A Scientific Statement from the American Association of Cardiovascular and Pulmonary Rehabilitation, the American Heart Association, and the American College of Cardiology. Circulation.

[114] Long L., Mordi I.R., Bridges C., Sagar V.A., Davies E.J., Coats A.J., Dalal H., Rees K., Singh S.J., Taylor R.S. (2019). Exercise-based cardiac rehabilitation for adults with heart failure. Cochrane Database Syst. Rev..

[115] Moser D.K., Arslanian-Engoren C., Biddle M.J., Chung M.L., Dekker R.L., Hammash M.H., Mudd-Martin G., Alhurani A.S., Lennie T.A. (2016). Psychological Aspects of Heart Failure. Curr. Cardiol. Rep..

[116] Amir O., Ben-Gal T., Weinstein J.M., Schliamser J., Burkhoff D., Abbo A., Abraham W.T. (2017). Evaluation of remote dielectric sensing (ReDS) technology-guided therapy for decreasing heart failure re-hospitalizations. Int. J. Cardiol..

[117] Boehmer J.P., Hariharan R., Devecchi F.G., Smith A.L., Molon G., Capucci A., An Q., Averina V., Stolen C.M., Thakur P.H. (2017). A Multisensor Algorithm Predicts Heart Failure Events in Patients with Implanted Devices: Results from the MultiSENSE Study. JACC Heart Fail..

[118] Alvarez-Garcia J., Lala A., Rivas-Lasarte M., De Rueda C., Brunjes D., Lozano-Jimenez S., Garcia-Sebastian C., Mitter S., Remior P., Jimenez-Blanco Bravo M. (2024). Remote Dielectric Sensing Before and After Discharge in Patients with ADHF: The ReDS-SAFE HF Trial. JACC Heart Fail..

[119] Izumida T., Imamura T., Koi T., Nakagaito M., Onoda H., Tanaka S., Ushijima R., Kataoka N., Nakamura M., Sobajima M. (2024). Prognostic impact of residual pulmonary congestion assessed by remote dielectric sensing system in patients admitted for heart failure. ESC Heart Fail..

[120] Abraham W.T., Adamson P.B., Bourge R.C., Aaron M.F., Costanzo M.R., Stevenson L.W., Strickland W., Neelagaru S., Raval N., Krueger S. (2011). Wireless pulmonary artery haemodynamic monitoring in chronic heart failure: A randomised controlled trial. Lancet.

[121] Codina P., Vicente Gomez J.A., Hernandez Guillamet G., Ricou Rios L., Carrete A., Vilalta V., Estrada O., Ara J., Lupon J., Bayes-Genis A. (2024). Assessing the impact of haemodynamic monitoring with CardioMEMS on heart failure patients: A cost-benefit analysis. ESC Heart Fail..

[122] Desai A.S., Bhimaraj A., Bharmi R., Jermyn R., Bhatt K., Shavelle D., Redfield M.M., Hull R., Pelzel J., Davis K. (2017). Ambulatory Hemodynamic Monitoring Reduces Heart Failure Hospitalizations in “Real-World” Clinical Practice. J. Am. Coll. Cardiol..

[123] Shavelle D.M., Desai A.S., Abraham W.T., Bourge R.C., Raval N., Rathman L.D., Heywood J.T., Jermyn R.A., Pelzel J., Jonsson O.T. (2020). Lower Rates of Heart Failure and All-Cause Hospitalizations During Pulmonary Artery Pressure-Guided Therapy for Ambulatory Heart Failure: One-Year Outcomes from the CardioMEMS Post-Approval Study. Circ. Heart Fail..

[124] Schmier J.K., Ong K.L., Fonarow G.C. (2017). Cost-Effectiveness of Remote Cardiac Monitoring with the CardioMEMS Heart Failure System. Clin. Cardiol..

[125] Abraham W.T., Stevenson L.W., Bourge R.C., Lindenfeld J.A., Bauman J.G., Adamson P.B., Group C.T.S. (2016). Sustained efficacy of pulmonary artery pressure to guide adjustment of chronic heart failure therapy: Complete follow-up results from the CHAMPION randomised trial. Lancet.

[126] Bristow M.R., Saxon L.A., Boehmer J., Krueger S., Kass D.A., De Marco T., Carson P., Di Carlo L., DeMets D., White B.G. (2004). Cardiac-resynchronization therapy with or without an implantable defibrillator in advanced chronic heart failure. N. Engl. J. Med..

[127] Cleland J.G., Daubert J.C., Erdmann E., Freemantle N., Gras D., Kappenberger L., Tavazzi L., Cardiac Resynchronization-Heart Failure (CARE-HF) Study Investigators (2005). The effect of cardiac resynchronization on morbidity and mortality in heart failure. N. Engl. J. Med..

[128] Poole J.E., Gleva M.J., Mela T., Chung M.K., Uslan D.Z., Borge R., Gottipaty V., Shinn T., Dan D., Feldman L.A. (2010). Complication rates associated with pacemaker or implantable cardioverter-defibrillator generator replacements and upgrade procedures: Results from the REPLACE registry. Circulation.

[129] Olde Nordkamp L.R., Brouwer T.F., Barr C., Theuns D.A., Boersma L.V., Johansen J.B., Neuzil P., Wilde A.A., Carter N., Husby M. (2015). Inappropriate shocks in the subcutaneous ICD: Incidence, predictors and management. Int. J. Cardiol..

[130] Weber D., Koller M., Theuns D., Yap S., Kuhne M., Sticherling C., Reichlin T., Szili-Torok T., Osswald S., Schaer B. (2019). Predicting defibrillator benefit in patients with cardiac resynchronization therapy: A competing risk study. Heart Rhythm.

[131] Canterbury A., Saba S. (2021). Cardiac resynchronization therapy using a pacemaker or a defibrillator: Patient selection and evidence to support it. Prog. Cardiovasc. Dis..

[132] Tang A.S., Wells G.A., Talajic M., Arnold M.O., Sheldon R., Connolly S., Hohnloser S.H., Nichol G., Birnie D.H., Sapp J.L. (2010). Cardiac-resynchronization therapy for mild-to-moderate heart failure. N. Engl. J. Med..

[133] Hadwiger M., Dagres N., Haug J., Wolf M., Marschall U., Tijssen J., Katalinic A., Frielitz F.S., Hindricks G. (2022). Survival of patients undergoing cardiac resynchronization therapy with or without defibrillator: The RESET-CRT project. Eur. Heart J..

[134] Gardner R.S., Singh J.P., Stancak B., Nair D.G., Cao M., Schulze C., Thakur P.H., An Q., Wehrenberg S., Hammill E.F. (2018). HeartLogic Multisensor Algorithm Identifies Patients During Periods of Significantly Increased Risk of Heart Failure Events: Results from the MultiSENSE Study. Circ. Heart Fail..

[135] Patel S.K., Luke A., Schutt A., Chinnadurai T., Pandya A., Saeed O., Goldstein D., Jorde U., Patel S.R. (2022). Evaluation of a novel virtual care platform for remote monitoring of LVAD patients. J. Heart Lung Transplant..

[136] Draper K.V., Huang R.J., Gerson L.B. (2014). GI bleeding in patients with continuous-flow left ventricular assist devices: A systematic review and meta-analysis. Gastrointest. Endosc..

[137] Hieda M., Sata M., Nakatani T. (2015). The Importance of the Management of Infectious Complications for Patients with Left Ventricular Assist Device. Healthcare.

[138] Tabit C.E., Chen P., Kim G.H., Fedson S.E., Sayer G., Coplan M.J., Jeevanandam V., Uriel N., Liao J.K. (2016). Elevated Angiopoietin-2 Level in Patients with Continuous-Flow Left Ventricular Assist Devices Leads to Altered Angiogenesis and Is Associated with Higher Nonsurgical Bleeding. Circulation.

[139] Tabit C.E., Coplan M.J., Chen P., Jeevanandam V., Uriel N., Liao J.K. (2018). Tumor necrosis factor-alpha levels and non-surgical bleeding in continuous-flow left ventricular assist devices. J. Heart Lung Transpl..

[140] Molina E.J., Shah P., Kiernan M.S., Cornwell W.K., Copeland H., Takeda K., Fernandez F.G., Badhwar V., Habib R.H., Jacobs J.P. (2021). The Society of Thoracic Surgeons Intermacs 2020 Annual Report. Ann. Thorac. Surg..

[141] Meineri M., Van Rensburg A.E., Vegas A. (2012). Right ventricular failure after LVAD implantation: Prevention and treatment. Best. Pract. Res. Clin. Anaesthesiol..

[142] Mehra M.R., Naka Y., Uriel N., Goldstein D.J., Cleveland J.C., Jr Colombo P.C., Walsh M.N., Milano C.A., Patel C.B., Jorde U.P. (2017). A Fully Magnetically Levitated Circulatory Pump for Advanced Heart Failure. N. Engl. J. Med..

[143] Mehra M.R., Goldstein D.J., Uriel N., Cleveland J.C., Jr Yuzefpolskaya M., Salerno C., Walsh M.N., Milano C.A., Patel C.B., Ewald G.A. (2018). Two-Year Outcomes with a Magnetically Levitated Cardiac Pump in Heart Failure. N. Engl. J. Med..

[144] Donze J., Aujesky D., Williams D., Schnipper J.L. (2013). Potentially avoidable 30-day hospital readmissions in medical patients: Derivation and validation of a prediction model. JAMA Intern. Med..

[145] Donze J.D., Williams M.V., Robinson E.J., Zimlichman E., Aujesky D., Vasilevskis E.E., Kripalani S., Metlay J.P., Wallington T., Fletcher G.S. (2016). International Validity of the HOSPITAL Score to Predict 30-Day Potentially Avoidable Hospital Readmissions. JAMA Intern. Med..

[146] Ibrahim A.M., Koester C., Al-Akchar M., Tandan N., Regmi M., Bhattarai M., Al-Bast B., Kulkarni A., Robinson R. (2020). HOSPITAL Score, LACE Index and LACE+ Index as predictors of 30-day readmission in patients with heart failure. BMJ Evid. Based Med..

[147] Obuobi S., Chua R.F.M., Besser S.A., Tabit C.E. (2021). Social determinants of health and hospital readmissions: Can the HOSPITAL risk score be improved by the inclusion of social factors?. BMC Health Serv. Res..

[148] Rajaguru V., Han W., Kim T.H., Shin J., Lee S.G. (2022). LACE Index to Predict the High Risk of 30-Day Readmission: A Systematic Review and Meta-Analysis. J. Pers. Med..

[149] van Walraven C., Wong J., Forster A.J. (2012). LACE+ index: Extension of a validated index to predict early death or urgent readmission after hospital discharge using administrative data. Open Med..

[150] Chamberlain R.S., Sond J., Mahendraraj K., Lau C.S., Siracuse B.L. (2018). Determining 30-day readmission risk for heart failure patients: The Readmission After Heart Failure scale. Int. J. Gen. Med..

[151] Regmi M.R., Parajuli P., Tandan N., Bhattarai M., Maini R., Garcia O.E.L., Bakare M., Kulkarni A., Robinson R. (2022). An assessment of race and gender-based biases among readmission predicting tools (HOSPITAL, LACE, and RAHF) in heart failure population. Ir. J. Med. Sci..

[152] Tan I.J., Barodi B., Buchan T.A., Kugathasan L., McDonald M., Ross H., Alba A.C. (2025). Guideline-Referral Criteria and Risk Profiles of Outpatients Referred to a Specialised Heart Failure Clinic. CJC Open.

[153] Allen L.A., Stevenson L.W., Grady K.L., Goldstein N.E., Matlock D.D., Arnold R.M., Cook N.R., Felker G.M., Francis G.S., Hauptman P.J. (2012). Decision making in advanced heart failure: A scientific statement from the American Heart Association. Circulation.

[154] Stewart G.C., Kittleson M.M., Patel P.C., Cowger J.A., Patel C.B., Mountis M.M., Johnson F.L., Guglin M.E., Rame J.E., Teuteberg J.J. (2016). INTERMACS (Interagency Registry for Mechanically Assisted Circulatory Support) Profiling Identifies Ambulatory Patients at High Risk on Medical Therapy After Hospitalizations for Heart Failure. Circ. Heart Fail..

